# Instrument for measuring home cooking skills in primary health care

**DOI:** 10.11606/s1518-8787.2022056003473

**Published:** 2022-08-16

**Authors:** Aline Rissatto Teixeira, Júlia Souza Pinto Camanho, Flavia da Silva Miguel, Helena Carvalho Mega, Betzabeth Slater

**Affiliations:** I Universidade de São Paulo Faculdade de Saúde Pública Programa de Pós-Graduação em Nutrição em Saúde Pública São Paulo SP Brasil Universidade de São Paulo. Faculdade de Saúde Pública. Programa de Pós-Graduação em Nutrição em Saúde Pública. São Paulo, SP, Brasil; II Universidade de São Paulo Faculdade de Saúde Pública Departamento de Nutrição São Paulo SP Brasil Universidade de São Paulo. Faculdade de Saúde Pública. Departamento de Nutrição. São Paulo, SP, Brasil

**Keywords:** Cooking, Knowledge, Psychometrics, instrumentation, Food and Nutrition Education, Primary Health Care

## Abstract

**OBJECTIVE:**

To develop and validate an instrument for measuring the home cooking skills of health professionals involved with guidelines for promoting adequate and healthy food in primary health care.

**METHODS:**

This is a methodological study with a psychometric approach, carried out in the city of São Paulo between January and November 2020, to develop and validate a self-applied online instrument. The data of the 472 participants were presented by descriptive statistics. Content validation was performed by expert judgment using the two round Delphi technique and empirical statistics for consensus evidence. Exploratory factor analysis was used for construct validation and reliability analysis, and the model adjustment rates and composite reliability were analyzed.

**RESULTS:**

The instrument presented satisfactory content validity for CVRc indices and 𝜅 in the two rounds of the Delphi technique. After the factor analysis, the final model of the Primary Health Care Home Cooking Skills Scale presented 29 items with adequate factorial loads (> 0.3). Bartlett’s and Kaiser-Meyer-Olkin’s (KMO) tests of sphericity performed in exploratory factorial analysis suggested interpretability in the correlation matrix, the parallel analysis indicated four domains and explained variance of 64.1%. The composite reliability of the factors was adequate (> 0.70) and the H-index suggested replicable factors in future studies. All adjustment rates proved to be adequate.

**CONCLUSIONS:**

The Primary Health Care Home Cooking Skills Scale presented evidence of validity and reliability. It is short and easy to apply and will make it possible to reliably ascertain the need for qualification of the workforce, favoring the planning of actions and public policies of promotion of adequate and healthy food in primary health care.

## INTRODUCTION

Home cooking skills (HCS) comprise actions such as menu planning, selection, mixing, cutting and cooking of food, the ability to perform tasks whilst cooking and confidence for culinary practices^1^. They are related to environmental and economic implications^2^ and are valued by the *Guia Alimentar para a População Brasileira* (Food Guidelines for the Brazilian Population)^3^ as an expression of cultural and social aspects. The document recognizes cooking as a strategic practice to promote adequate and healthy food (AHF), aiming to reduce the choice of ultra-processed foods, which are associated with overweight, obesity, cancer and other diseases^4,5^. Therefore, recognition of cooking should be paramount in food and nutrition education actions^2^.

In Brazil, the guidelines of AHF are located substantially within the scope of primary health care (PHC), the first level of care and link of subjects with the Unified Health System. PHC professionals play a relevant role in promoting food and nutrition education actions involving culinary practices, such as dissemination of recipes, workshops, guided visits to open-air markets, home visits and sensory exploration of food^6^. Such actions allow health workers to convey technical knowledge to the daily lives of the subjects, therefore it is important that those workers develop their home cooking skills^7^.

An accurate diagnosis of these skills is essential to promote workforce qualification and plan public health actions and policies on the subject, and it relies on the use of valid and reliable instruments, based on robust psychometric criteria^8,9^. Teixiera et al.^10^ identified and critically analyzed the psychometric quality of 12 Brazilian and international instruments for measuring cooking skills in adults. The psychometric attributes of those instruments were considered insufficient, with unsatisfactory results based on statistical criteria or methodological inadequacies. Two of the studies were Brazilian: Jomori^11^ performed a cross-cultural adaptation of an instrument based on the program *Cooking with a Chef*, from Clemson University. The results of a part of the scale of this instrument were unsatisfactory for reliability. Martins et al.^12^ developed a cooking confidence scale for parents of schoolchildren. The authors evaluated the internal consistency, stability and content validity of the instrument, but did not report agreement rates between experts and procedures for construct validity.

Thus, there is a strong need to develop a new instrument for assessing home cooking skills aimed at Brazilian health professionals involved with guidelines for promoting adequate and healthy food in PHC, based on psychometric criteria that follow methodological rigor to determine its validity and reliability recommended in the scientific literature.

## METHODS

This is a methodological study with a psychometric approach^13^ conducted between January and November 2020.

This study was approved by the research ethics committee of University of São Paulo (CAAE 15194819.8.0000.5421, No. 3,502,315) and by the co-participating institution of the São Paulo Municipal Health Department (SMS-SP) (No. 3,585,369). The participants were informed of the objectives of the study and confidentiality of the data through an informed consent form.

In the prototype stage, a working group was created with nine members of both sexes and from different Brazilian states (São Paulo, Mato Grosso, Pará and Minas Gerais). They were nutrition and gastronomy majors from the Faculdade de Saúde Pública of Universidade de São Paulo (FSP-USP) involved with culinary approach disciplines and PhD researchers with experience in the elaboration and validation of research instruments to systematically develop the instrument.

To define the theoretical domains and items of the first version of the instrument proposed in this study, the following were considered: (a) professional experience and culinary experience of the group; (b) exploration of theoretical framework on HCS^14^(c) systematic review to identify and analyze psychometric properties of instruments that assessed the home cooking skills of adults^10^. The domains, items and response formats of the instruments identified in this review were discussed by the research group for the construction of the prototype.

The construction of the initial set of items and response formats of the prototype version of the instrument, entitled Primary Health Care Home Cooking Skills Scale (PHCHCSS), followed the quality recommendations proposed by DeVellis^15^.

The next phase, the psychometric phase, consisted of three stages. The first stage featured experts from various professional levels, including university professors, researchers and nutrition and gastronomy professionals from Brazil^16^. A number of participants between three and 10 was considered sufficient^17^.

The two-round Delphi method ^18^ was used. Experts completed online questionnaires, with semi-structured questions of sociodemographic characterization and evaluation of the items and theoretical domains of the instrument built in the prototype phase. They proposed improvements, inclusion and exclusion of items, adequacy of the options of the instrument scale, and responded to a scale Likert of agreement (1 = strongly disagree and 4 = strongly agree) to evaluate each item for:

- Clarity: Was the item written in such a way that the concept is understandable and adequately expresses what is to be measured?
- Pertinence: Does the item reflect the concepts involved in the domain and is it adequate to achieve the proposed objectives?
- Relevance: Is the item important for the construction of the domains that are the focus of the research scale?

The first round of the panel took place between March 26 and April 29, 2020 and featured eight experts. The research group assessed the comments provided, excluded irrelevant and non-pertinent items, made adjustments to those considered unclear and included suggested items for a better coverage of the phenomenon. The instrument was re-submitted to the experts for evaluation after the modifications. Second round, started on May 28, 2020, lasted 30 days and featured seven experts.

The characteristics of the study participants were presented by descriptive statistics. The Critical Content Validity Ratio - CVRc was used to statistically analyze the validity of each attribute of the items and domains^19^and the Kappa coefficient (k) was calculated to evaluate the agreement between experts on each item^20^ of the two rounds of the panel. Items with CVRc > 0.05 and k ≥ 0,60^20^ were retained^19^. The content validity index (CVI) was also used to analyze the validity of the instrument as a whole^21^. The result > 0.8 was considered acceptable^22^.

The second stage was the pre-test phase, in which professionals from a health center in the city of São Paulo, with similar characteristics to the research population of the project, tested the usability of PHCHCSS. The pre-test participants were not part of the construct validity sample and reliability analysis of the instrument. They commented on possible difficulties in filling out the instrument, clarity and adequacy of the questions to the objective of the research and recorded response times.

In the third stage, construct validity and reliability of PHCHCSS were tested. The scale was developed for professionals involved in the promotion of adequate and healthy nutrition in basic health units (BHU) of São Paulo’s Municipal Health Department (SMS-SP). There are 464 BHU in the city of São Paulo^23^.

The sample included professionals who expressed their consent to participate. Recruitment was done by contacting regional health coordinators, technical health supervisors and BHU managers to collect the emails addresses of target professionals. A website was also developed^a^, advertised on social media to present and clarify the purpose of the research and to recruit participants. The number of participants in the sample was based on the recommendations of Costello and Osborne^24^, of 10 subjects per instrument item.

Data collection began on August 2, 2020, lasting 30 days. A total of 472 professionals answered a sociodemographic questionnaire and PHCHCSS online. Their characteristics were presented by descriptive statistics.

Exploratory factor analysis (EFA) was used to evaluate the factorial structure of the PHCHCSS. Polychoric correlation and the Robust Diagonally Weighted Least Squares (RDWLS) extraction method were used. The decision on the number of retained factors was made by parallel analysis with random permutation of the observed data^25^. The rotation used was the Robust Promin^26^. Values of 60% of the total variance explained, items with commonality ≥ 0.4 and factorial loads ≥ 0.30 were considered satisfactory. Items with cross-factorial loads were excluded^27^. KMO values ≥ 0.70 and significant values for Bartlett’s index represented adequacy measures of the sample^28^.

The goodness of fit of the model was evaluated using the Root Mean Square Error of approximation (RMSEA) index, the Comparative Fit Index (CFI) and the Tucker-Lewis Index (TLI). RMSEA values should be < 0.08, and CFI and TLI values should be > 0.90 or, preferably, 0.95^29,30^.

The stability of the factors was assessed by the H-index, which assesses how well a set of items represents a factor. H values > 0.80 suggest a well-defined and probably stable latent variable in different studies^31^.

To test the reliability, the composite reliability (CR) was calculated, with acceptable values > 0.70^32^.

All statistical analyses were performed using the statistical software Factor, version 10.10.03^29^.

## RESULTS

The Box presents details on the theoretical domains and the construction of a set of items of the prototype version of the PHCHCSS based on the discussions of the research group, exploration of the theoretical framework and systematic review. Forty-four items were proposed to evaluate the home cooking skills of PHC professionals, with response options structured into a five-point Likert-type scale (0 = strongly disagree and 5 = strongly agree).

**Box:** Identification of theoretical domains and construction of a set of items of the prototype version of the PHCHCSS.

Theoretical domains	Reference	Labels/items	Reference
1. Shopping planning and meal preparation	BRASIL^3^ (2014); Lavelle et al.^34^ (2017); Ternier^36^ (2010); Kennedy et al. (2019)^a^	*When planning my shopping and meals, I perform the following tasks satisfactorily:*	
		1. Research the harvest year when buying fruits, vegetables and legumes	Adapted from: Lavelle et al.^34^ (2017)
		2. Make a shopping list before going to the supermarket	Adapted from: Lavelle et al.^34^ (2017); Kennedy et al. (2019)^a^
		3. Research food prices before buying them	Adapted from: Lavelle et al.^34^ (2017); Kennedy et al. (2019)^a^
		4. Buy food in food markets	From the authors
		5. Organize myself to prepare the meals I will consume throughout the week	Adapted from: Lavelle et al.^34^ (2017); Kennedy et al. (2019)^a^
		6. Freeze prepared meals in batches to reduce time in the kitchen	Adapted from: Lavelle et al.^34^ (2017)
2. Culinary creativity	Lavelle et al.^34^ (2017); Mills et al.^38^ (2017); Short (2006)^b^; Michaud (2007)^c^; Jomori et al. (2017)^d^	*I consider myself creative enough to:*	
		7. Cook different preparations from the same ingredients	From the authors
		8. Create different salad dressings	From the authors
		9. Use leftovers to prepare a new meal	Adapted from: Lavelle et al.^34^ (2017); Michaud (2007)^c^; Jomori et al. (2017)^d^
		10. Use unconventional parts of food (e.g., leaves, skin, stalks, seeds) to prepare recipes	From the authors
3. Preparation and multitasking skills	Martins et al.^12^ (2019); Lavelle et al.^34^ (2017); Ternier^36^ (2010); Mills et al.^38^ (2017); Kennedy et al. (2019)^a^; Short (2006)^b^ Hartmann et al. (2013)^e^; Kowalkowska et al. (2018)^f^; Vhrovnik (2012)^g^	*I believe that I have enough skills to:*	
		11. Blanch broccoli florets, applying heat shock for the appropriate time	From the authors
		12. Briefly soak beans in hot water, discarding the water after 1 hour	From the authors
		13. Chop an onion properly into small cubes	Adapted from: Kennedy et al. (2019)^a^
		14. Prepare vegetable stock from fresh ingredients	From the authors
		15. Check if a cake has finished baking with a wooden stick	Adapted from Martins et al.^12^ (2019); Hartmann et al. (2013) and; Kowalkowska et al. (2018)^f^
		16. Thicken starch-based preparations without forming lumps	From the authors
		17. Measure the correct amount of water to prepare fluffly rice	From the authors
		18. Quickly desalt dried meat in boiling water	From the authors
		19. Butcher a chicken by myself	From the authors
		20. Correct the acidity of sauces using *in natura* ingredients, like carrots	From the authors
		21. Prepare homemade tomato sauce	Adapted from Martins et al.^12^ (2019); Hartmann et al. (2013) and; Kowalkowska et al. (2018)^f^
		22. Tenderize tough meats, like beef shank, by stewing	From the authors
		23. Prepare a homemade feijoada from scratch	From the authors
		24. Fry potatoes properly, without making them greasy	Adapted from: Lavelle et al.^34^ (2017); Kennedy et al. (2019)^a^ Vhrovnik (2012)^g^
		25. Cook while doing other household chores (e.g., laundry, house cleaning)	From the authors
		26. Attend to a matter over the phone while cooking pasta	From the authors
		27. Prepare a main meal (lunch/dinner) in less than 30 minutes	Adapted from: Lavelle et al.^34^ (2017)
4. Sensory perception	Lavelle et al.^34^ (2017); Mills et al.^38^ (2017); Short (2006)^b^; Vhrovnok (2012)^g^	*I consider my sensory perceptions suitable to:*	
		28. Replace fresh herbs with dried herbs in cooking preparations by using only my sensory perceptions	Adapted from: Lavelle et al.^34^ (2017); Kennedy et al. (2019)^a^
		29. Dose the amount of spices when experimenting with food during preparation	From the authors
		30. Combining foods based on previous cooking experiences	From the authors
		31. Judge that a meal based on pasta with tomato sauce, watermelon juice and strawberry jelly has inadequate visual appeal	From the authors
		32. Differentiate sauces prepared with industrialized vegetable stock from those prepared with natural ingredients by using only my palate	From the authors
		33. Identify the doneness of grilled meat (rare, medium, well done) by using only my perceptions of texture (e.g., by pressing it with a spatula)	Adapted from Vhrovnok (2012)^g^
		34. Know when the flour used to prepare a white sauce is cooked properly by using only the sense of smell (to identify the almond aroma)	From the authors
5. Trust	Martins et al.^12^ (2019); Lavelle et al.^34^ (2017); Short (2006)^b^; Michaud (2007)^c^; Jomori et al. (2017)^d^; Hartmann et al. (2013)^e^; Kowalkowska et al. (2018)^f^ Barton et al. (2011)^h^	*I am confident enough to:*	
		35. Use the pressure cooker alone	Adapted from Martins et al.^12^ (2019)
		36. Prepare a caramel sauce for a flan	From the authors
		37. Follow a recipe from start to finish	Adapted from Michaud (2007)^c^; Jomori et al. (2017)^d^; Barton et al. (2011)^h^
		38. Bake homemade bread by myself	Adapted from: Hartmann et al. (2013)and; Kowalkowska et al (2018)^f^
		39. Grill meat to the desired doneness	From the authors
		40. Roast a whole bird	Adapted from: Lavelle et al.^34^ (2017)
		41. Adjust the amount of ingredients in a recipe for a larger number of people	From the authors
		42. Convert universal measurements (gram, kilogram, liter) into homemade measurements (spoonful, glass, cup)	From the authors
		43. Bake a simple cake without a recipe	Adapted from Martins et al.^12^ (2019); Hartmann et al. (2013) and; Kowalkowska et al. (2018)^f^
		44. Handle unexpected situations when cooking (e.g., turning overwhipped cream into butter)	From the authors

PHCHCSS: Primary Health Care Home Cookin Skills Scale.

^a^ Kennedy LG, Kichler EJ, Seabrook JA, Matthews JI, Dworatzek PDN. Validity and Reliability of a Food Skills Questionnaire. J Nutr Educ Behav. 2019;51(7):857-864. DOI: https://doi.org/10.1016/j.jneb.2019.02.003

^b^ Short F. Kitchen Secrets: Berg Publishers; 2006.168 p

^c^ Michaud P. Development and evaluation of instruments to measure the effectiveness of a culinary and Nutrition education program. Thesis. Clemson: Clemson University, SC. 2007

^d^ Jomori, MM; Proença, RPdaC; Echevarria-Guanilo, ME; Bernardo, GL; Uggioni, PL; Fernandes, AC. Construct validity of Brazilian cooking skills and healthy eating questionnaire by the known-groups method. Br Food J. 2017, 119(5)00-00. DOI: http://dx.doi.org/10.1108/BFJ-10-2016-0448

^e^ Hartmann C, Dohle S, Siegrist M.Importance of cooking skills for balanced food choices. Appetite. 2013; 65, 125-31. DOI: https://doi.org/10.1016/j.appet.2013.01.016

^f^ Kowalkowska J, Poı´nhos R; Rodrigues S. Cooking skills and socio-demographics among Portuguese university students. Br Food J. 2018, 120(3)563–577.DOI: https://doi.org/10.1108/BFJ-06-2017-0345

^g^ Vrhovnik L. A pilot study for the development of a food skills survey tool. Dissertation. Queen’s University. Kingston, Ontario, Canada, 2012. DOI: https://doi.org/10.1007/s10653-011-9439-6

^h^ Barton KL, Wrieden WL, Anderson AS. Validity and reliability of a short questionnaire for assessing the impact of cooking skills interventions. J Hum Nutr Diet. 2011; 24, 588–595. DOI: https://doi.org/10.1111/j.1365-277X.2011.01180.x

The instruments identified in a systematic review had dimensions of planning, selection and purchase of food and confidence in food preparation, and may or may not include pre-prepared and convenience products.

For the PHCHCSS, the theoretical dimensions of HCS for the construction of the initial items were considered to be the food shopping planning and meal preparation, culinary creativity, the use of sensory perception and confidence in the preparation of meals based on fresh, minimally processed and culinary ingredients, as recommended by the *Guia Alimentar para a População Brasileira*. Multitasking skills were also identified as a theoretical domain. They are defined in the scientific literature as the ability to perform tasks simultaneously in the home environment, representing an advantage when preparing meals.

The prototype version of the instrument was submitted to content evaluation by experts. The main results of the development and validation of the PHCHCSS are shown in the Figure.

**Figure:**
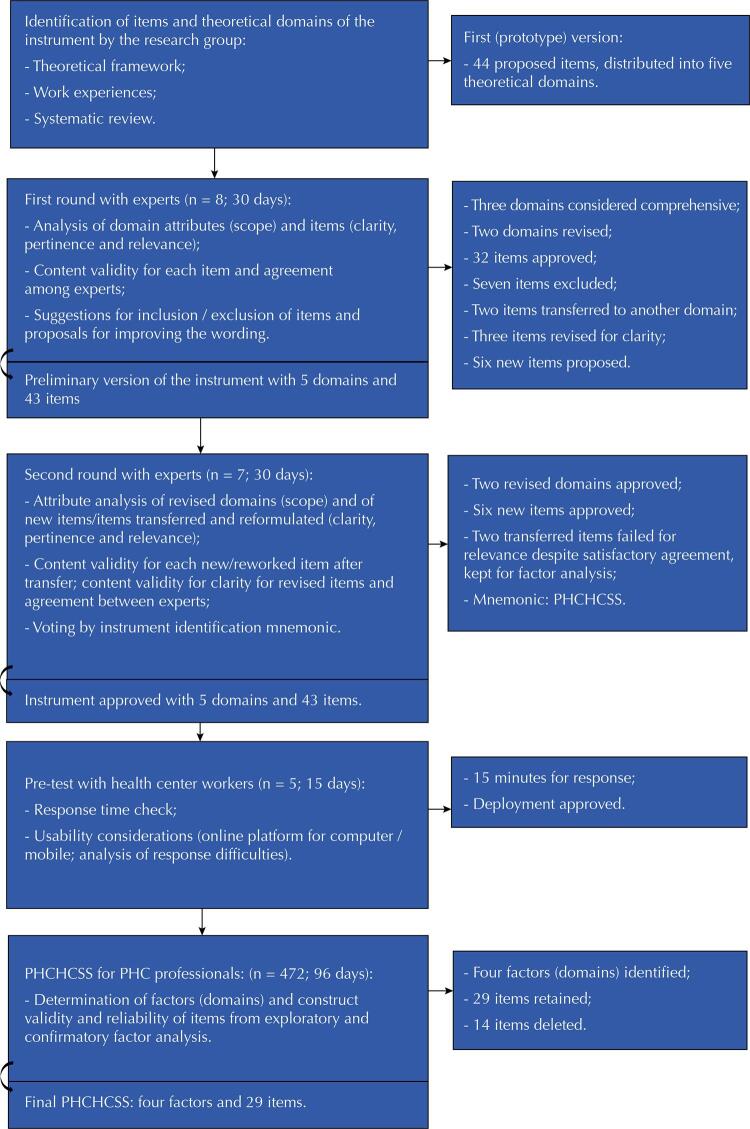
Flowchart of the development process of the items and domains of the instrument.

PHCHCSS: Primary Health Care Home Cooking Skills Scale; PHC: primary health care.

In the psychometric phase, the first stage was the validation of the content. The study sample size was adequate for this stage. The response rate for the first round of the Delphi technique was 72.7% (8/11) and 87.5% (7/8) for the second round. Most of the experts were female (n = 7; 87.5%), with a mean age of 42.3 years (SD = 9.0). Of the total, 37.5% (n = 3) were experts (*latu sensu*), 12.5% (n = 1) masters, 25% (n = 2) PhDs and 12.5% (n = 1) full professors. The panel also had a lay participant (n = 1; 12.5%) with training in gastronomy and a full-time job cooking. The experts were professors at public (25%) and private (12.5%) universities, researchers (12.5%), nutritionists in food services (37.5%) and culinary professionals (12.5%). The length of professional experience ranged from 10 to 33 years (mean = 17.8 years; SD = 7.9 years). The average time devoted to culinary practices among experts was 12.2 hours per week (SD = 9.6 hours per week).

The evaluation of the experts resulted in the exclusion of seven items from the prototype version of the instrument, two items transferred from their original domains, three items revised for clarity, six new items proposed and a change from the agreement scale to a frequency scale, totaling 43 valid items for content. An overview of the content validity analysis of the instrument is shown in Table 1.

**Table 1:** Content validity analysis of the Primary Health Care Home Cooking Skills by experts.

Round 1: 8 experts								
Scope^a^	κ^b^ (%)	Action	Item^c^	Clarity^a^	Pertinence^a^	Relevance^a^	κ^b^ (%)	Action
**Domain 1. Shopping planning and meal preparation** When planning my shopping and meals, I satisfactorily perform the following tasks:								
0.5	**0.14 (57.1)**	Add items	Research the harvest year when buying fruits, vegetables and legumes	0.75	1	1	0.83 (91.7)	Valid
			Make a shopping list before going to the supermarket	0.75	1	1	0.83 (91.7)	Valid
			Research food prices before buying them	0.75	1	0.75	0.67 (83.3)	Valid
			Buy food in food markets	0.75	0.75	0.75	0.5 (75.0)	Deleted by the author^d^
			Organize myself to prepare the meals I will consume throughout the week	0.75	1	1	0.83 (91.7)	Valid
			Freeze prepared meals in batches to reduce time in the kitchen	**0.5**	1	1	0.71 (85.7)	Review clarity
**Domain 2. Cooking creativity** I consider myself creative enough to:								
0.5	**0.14 (57.1)**	Add items	Cook different preparations from the same ingredients	1	1	1	1 (100)	Valid
			Create different salad dressings	1	1	0.75	0.83 (91.7)	Valid
			Use leftovers to prepare a new meal	1	1	1	1 (100)	Valid
			Use unconventional parts of food (e.g., leaves, skin, stalks, seeds) to prepare recipes	**0.5**	0.75	0.75	**0.38** (69.5)	Transfer to D1 w / change
**Domain 3. Preparation and multitasking skills** I believe that I have enough skills to:								
1	1 (100)	Comprehensive domain	Blanch broccoli florets, applying heat shock for the appropriate time	**0.25**	0.75	**0.5**	**0.19 (59.5)**	Deleted by panel
			Briefly soak beans in hot water, discarding the water after 1 hour	**0.25**	**0.5**	**0.5**	**0.07 (53.6)**	Deleted by panel
			Chop an onion properly into small cubes	1	1	**0.25**	0.64 (82.1)	Deleted by panel
			Prepare vegetable stock from fresh ingredients	1	1	1	1 (100)	Valid
			Check if a cake has finished baking with a wooden stick	1	1	1	1 (100)	Valid
			Thicken starch-based preparations without forming lumps	0.75	1	1	0.83 (91.7)	Valid
			Measure the correct amount of water to prepare fluffly rice	1	1	1	1 (100)	Valid
			Quickly desalt dried meat in boiling water	0.75	0.75	0.75	0.5 (75.0)	Deleted by the author^d^
			Butcher a chicken by myself	1	1	0.75	0.83 (91.7)	Valid
			Correct the acidity of sauces using *in natura* ingredients, like carrots	1	1	1	1 (100)	Valid
			Prepare homemade tomato sauce	1	1	1	1 (100)	Valid
			Tenderize tough meats, like beef shank, by stewing	1	0.75	0.75	0.67 (83.3)	Valid
			Prepare a homemade feijoada from scratch	1	1	0.75	0.83 (91.7)	Deleted by the author^d^
			Fry potatoes properly, without making them greasy	1	0.75	1	0.83 (91.7)	Valid
			Cook while doing other household chores (e.g., laundry, house cleaning)	1	1	1	1 (100)	Valid
			Attend to a matter over the phone while cooking pasta	1	0.75	0.75	0.67 (83.3)	Valid
			Prepare a main meal (lunch/dinner) in less than 30 minutes	0.75	1	1	0.83 (91.7)	Valid
**Domain 4. Sensory perception** I consider my sensory perceptions suitable to:								
**0.75**	0.5 (75)	Comprehensive domain	Replace fresh herbs with dried herbs in cooking preparations by using only my sensory perceptions	0.75	1	1	0.83 (91.7)	Valid
			Dose the amount of spices when experimenting with food during preparation	0.75	1	1	0.83 (91.7)	Valid
			Combining foods based on previous cooking experiences	1	0.75	1	0.83 (91.7)	Valid
			Judge that a meal based on pasta with tomato sauce, watermelon juice and strawberry jelly has inadequate visual appeal	**0.25**	**0.5**	**0.5**	**0.07 (53.6)**	Deleted by panel
			Differentiate sauces prepared with industrialized vegetable stock from those prepared with natural ingredients by using only my palate	1	1	1	1 (100)	Valid
			Identify the doneness of grilled meat (rare, medium, well done) by using only my perceptions of texture. (e.g., by pressing it with a spatula)	0.75	1	1	0.83 (91.7)	Valid
			Know when the flour used to prepare a white sauce is cooked properly by using only the sense of smell (to identify the almond aroma)	**0.5**	0.75	0.75	**0.38 (69.5)**	Review clarity
**Domain 5. Confidence** I am confident enough to:								
**1**	1 (100)	Comprehensive domain	Use the pressure cooker alone	1	1	1	1 (100)	Valid
			Prepare a caramel sauce for a flan	1	1	0.75	0.83 (91.7)	Valid
			Follow a recipe from start to finish	1	1	1	1 (100)	Valid
			Bake homemade bread by myself	1	1	1	1 (100)	Valid
			Grill meat to the desired doneness	**0.5**	1	1	0.71 (85.7)	Review clarity
			Roast a whole bird	1	1	1	1 (100)	Valid
			Adjust the amount of ingredients in a recipe for a larger number of people	1	1	1	1 (100)	Valid
			Convert universal measurements (gram, kilogram, liter) into homemade measurements (spoonful, glass, cup)	0.75	1	1	0.83 (91.7)	Valid
			Bake a simple cake without a recipe	1	1	1	1 (100)	Valid
			Handle unexpected situations when cooking (e.g., turning overwhipped cream into butter)	1	1	1	1 (100)	Transferir para D2 s/ alteração
CVI instrument (Lawshe, 1975)								0.83
**Round 2: 7 experts**								
**Scope^a^**	**κ^b^ (%)**	**Action**	**Item^c^**	**Clarity^a^**	**Pertinence^a^**	**Relevance^a^**	**κ^b^ (%)**	**Action**
**Domain 1. Shopping planning and meal preparation** I perform the following actions:								
**1**	1 (100)	Comprehensive domain	Consider the harvest year when buying fruits, vegetables and legumes	-	-	-	-	Valid in Round 1
			Make a list before I go shopping	-	-	-	-	Valid in Round 1
			Consider food prices before buying it	-	-	-	-	Valid in Round 1
			Organize my time to produce the meals I will consume throughout the week	-	-	-	-	Valid in Round 1
			Freeze prepared meals in batches to consume on other days	1	-	-	1 (100)	Maintained/valid
			Plan the menu considering the use of unconventional parts of food (e.g., skins, stalks, seeds)	1	1	**0.71**	0.81 (90.5)	Maintained^e^
			Decide the amount of food to be bought based on the number of people eating at home^f^	1	1	1	0.81 (90.5)	Valid
			Plan the meals I will consume taking into account the variety of foods (e.g., vegetables, legumes, meats, grains)^f^	1	1	1	1 (100)	Valid
			Check the items I have at home before buying food^f^	1	1	1	1 (100)	Valid
**Domain 2. Cooking creativity** I use my creativity to:								
**1**	1 (100)	Comprehensive domain	Prepare different recipes from the same ingredients	-	-	-	-	Valid in Round 1
			Create different sauces to vary meals	-	-	-	-	Valid in Round 1
			Use leftovers from previous meals to prepare a new recipe	-	-	-	-	Valid in Round 1
			Adapt recipes with the ingredients I have at home^f^	1	1	1	1 (100)	Valid
			Handle unexpected situations when I’m cooking (e.g., preparing a soup with overcooked beans)^f^	1	1	**0.71**	0.81 (90.5)	Maintained^e^
**Domain 3. Preparation and multitasking skills** I perform the following actions:								
**1**	1 (100)	Comprehensive domain	Prepare stocks from ingredients *in natura* (e.g., fresh vegetables, meat trimmings) to impart flavor to culinary preparations	-	-	-	-	Valid in Round 1
			Recognize when a cake is fully baked	-	-	-	-	Valid in Round 1
			Thicken cooking preparations with cornstarch without forming lumps	-	-	-	-	Valid in Round 1
			Use the correct amount of water to prepare fluffy rice	-	-	-	-	Valid in Round 1
			Cut a chicken into pieces	-	-	-	-	Valid in Round 1
			Correct the acidity of sauces using *in natura* ingredients, like carrots	-	-	-	-	Valid in Round 1
			Prepare homemade tomato sauce	-	-	-	-	Valid in Round 1
			Cook tough meats, such as beef shank, in liquid to make them softer	-	-	-	-	Valid in Round 1
			Fry food without making it greasy	-	-	-	-	Valid in Round 1
			Do other household chores (e.g., laundry, house cleaning) while cooking	-	-	-	-	Valid in Round 1
			Attend to a matter on the phone while the pots are on the heat	-	-	-	-	Valid in Round 1
			Prepare lunch / dinner from scratch in less than 30 minutes	-	-	-	-	Valid in Round 1
			Discard the water in which the beans soaked before cooking them	1	1	1	1 (100)	Valid
**Domain 4. Sensory perception** I use my sensory perceptions to:								
		Domain considered comprehensive in Round 1	Replacing fresh herbs with dried herbs in culinary preparations by using only my sensory perceptions	-	-	-	-	Valid in Round 1
			Adjust the amount of seasonings when tasting food during preparation	-	-	-	-	Valid in Round 1
			Combine foods based on their taste	-	-	-	-	Valid in Round 1
			Differentiate sauces prepared with industrialized vegetable stock from those prepared with natural ingredients by using only my palate	-	-	-	-	Valid in Round 1
			Identify how cooked food is according to its consistency (e.g., hard, soft, mushy)	-	-	-	-	Valid in Round 1
			Recognize that a white sauce is ready according to its thick texture	1	-	-	1 (100)	Maintained/valid
			Identify whether foods are suitable for consumption based on their sensory characteristics (e.g., red color of strawberries, soft texture of avocados, sour smell of spoiled food)	1	1	1	1 (100)	Valid
**Domain 5. Confidence** I feel confident to:								
		Domain considered comprehensive in Round 1	Use the pressure cooker alone	-	-	-	-	Valid in Round 1
			Prepare a sugar syrup	-	-	-	-	Valid in Round 1
			Follow a cooking recipe from start to finish	-	-	-	-	Valid in Round 1
			Bake homemade bread	-	-	-	-	Valid in Round 1
			Cook food according as instructed in a recipe (e.g., firm beans for a salad, medium meats)	1			1 (100)	Maintained/valid
			Roast a whole bird	-	-	-	-	Valid in Round 1
			Adjust the amount of ingredients in a recipe for a larger number of people	-	-	-	-	Valid in Round 1
			Convert universal measurements (e.g., gram, kilo, litre) to home measurements (e.g., spoonful, glass, cup)	-	-	-	-	Valid in Round 1
			Bake a simple cake without instructions	-	-	-	-	Valid in Round 1
CVI instrument (Lawshe, 1975)								0.97

CVI: content validity index.

^a^ Round 1: CVRc: 8 experts = p = 0.05 = 0.693; Round 2: CVRc: 7 experts = p = 0.05 = 0.741^19^.

^b^ The Kappa (k) coefficient for agreement among experts was performed with the help of the calculator available at: http://justusrandolph.net/kappa/.

^c^ Initial response scale of the instrument corresponded to the Likert type for agreement, as follows: (1) Strongly disagree, (2) disagree, (3) Neither agree nor disagree, (4) Agree, (5) Strongly agree. This response scale was changed to a frequency scale at the suggestion of the experts after Round 1. Experts reported that the respondents found it difficult to understand the agreement scales. In addition, agreement scales can indicate the individual’s perception of their skills, but they do not necessarily reflect the behaviors that make it possible to actually measure such skills. In this case, the adequacy for a frequency scale was shown to be more consistent with the objective of this instrument. The amendment was approved by the experts who took part in Round 2. The new scale is presented as follows: (1) Never, (2) Almost Never, (3) Sometimes, (4) Almost always, (5) Always. The authors made minimal changes to the labels and questions of the instrument (without changing their objectives) to make them coherent with the new response scale. Those changes were also approved by the experts.

^d^ Items excluded by the researchers with the consent of the expert group after consultation in the second stage of the panel. The item *Buy food at food markets* was excluded considering that one can shop for food in other places, not only food markets, and that this subject was included in other items of the instrument; item *Quickly desalt dried meat in boiling water* was excluded considering that the purpose of the instrument is to measure the cooking skills for preparing daily meals (dried meat is not consumed on a daily basis in the city of São Paulo) and, considering the frequency label, there could be an interpretation bias on the part of respondents (respondents could inform the frequency with which they prepare dried meat without desalting it); *Prepare homemade feijoada from the scratch* was excluded considering that the purpose of the instrument is to measure cooking skills for preparing everyday meals (feijoada, though a typical Brazilian dish, is not prepared every day in the city of São Paulo) and, considering the frequency label, there could be an interpretation bias on the part of respondent (respondents could inform the frequency with which they cook feijoada and not if they make it from scratch).

^e^ Justification not presented by the expert or not accepted by the author. Although the CVRc were slightly lower than the critical reference limit for the relevance attribute^19^, the item was kept in view of the value of agreement among experts on the item and to ensure the coverage of the domain. We chose to keep the item and investigate its behavior in factor analysis.

^f^ New items, submitted to content validity analysis (clarity, pertinence, relevance and agreement among experts) in the second Round of the panel of experts. The themes included in the added items comprised suggestions from experts in the first Round, to expand the scope of the domains.

The second stage, pre-test, was conducted by five professionals from a health center in the city of São Paulo. The covid-19 pandemic posed an obstacle for recruitment, given the intensified demand for care at BHU. The sample was composed of women who worked as nutritionists (n = 3; 60%), psychologists (n = 1, 20%) and nurses (n = 1; 20%). This sample was not part of the validity and reliability analysis of the instrument. Participants reported that the instrument was easy to access by computer, comprehension of the questions and answer options, with a suggestion to enlarge the font size of the questions, which was adopted by the research group. The average response time was 15 minutes.

The third stage consisted in performing construct validity and reliability analysis. The study sample size was adequate for this stage. Table 2 shows the characteristics of the 472 health professionals from the participating PHC.

**Table 2:** Characteristics of health professionals participating in the study of construct validity and reliability of the Primary Health Care Home Cooking Skills Scale.

Variable	PHC professionals
	BHU (n = 472)
	Average (SD) [%]
CRS-SMS/SP	
North	[9%]
South	[38%]
Southeast	[11%]
Centre	[3%]
East	[33%]
West	[7%]
Age (years)	38 (9)
Race/color	
White	[48%]
Yellow	[3%]
Brown	[33%]
Black	[15%]
Sex	
Female	[90%]
Male	[10%]
Gender	
Cisgender woman	[83%]
Transgender woman	[1%]
Cisgender man	[9%]
Not declared	[7%]
Marital status	
Single(a)	[36%]
Married	[45%]
Domestic partnership	[11%]
Divorced	[7%]
No. Household/dependent residents	
Lives alone	[9%]
One	[25%]
Two	[28%]
Three	[23%]
Four	[8%]
Five	[6%]
Six or more	[1%]
Average household income (minimum wages)^a^	
None	[1%]
< 1	[2%]
1–3	[29%]
3–6	[22%]
6–9	[19%]
9–12	[11%]
12–15	[7%]
> 15	[9%]
Profession/field	
Nursing	[33%]
Community health worker	[25%]
Medicine	[10%]
Nutritionist	[10%]
Dentistry/oral health	[5%]
Pharmacy	[4%]
Others^b^	[9%]
Schooling	
High school	[21%]
Technical school	[12%]
Bachelor’s degree	[14%]
Post-graduate (*Lato sensu*)	[48%]
Post-graduate *(Stricto sensu*)	[5%]
Consultation time (min)	
≤ 15	[35%]
16–30	[42%]
31–45	[16%]
46–60	[5%]
≥ 61	[2%]
Training in gastronomy/cooking	
Yes	[3%]
No	[97%]
Previous work experience with cooking	
Yes	[15%]
No	[85%]
Interest in PHC cooking skills	
Yes	[90%]
No	[10%]
Guides cooking skills in PHC	
Always	[28%]
Sometimes	[60%]
Never	[12%]
Sources on cooking skills^c^	
None	[2%]
Family	[49%]
Books	[44%]
Magazines/internet/shows	[77%]
Free courses/vocational training	[15%]
Matrix support/BHU nutritionist/Continuing Education at BHU/ Food Guidelines	[6%]
Others^d^	[1%]
Distance run to buy FVG (km)	
≤ 1	[44%]
1–2	[26%]
2–3	[15%]
3–4	[5%]
4–5	[4%]
> 5	[6%]
Buys food for the home	
Yes	[86%]
No	[14%]
Meals outside the home (at home/week)	
0	[11%]
< 2	[49%]
2–4	[22%]
4–6	[13%]
> 6	[6%]
Time spent cooking (hours/week)	6:25 (7:29)
Degree of knowledge [GAPB; ARB; CFLV]	
None	[47%; 70%; 72%]
Low	[24%; 18%; 15%]
Reasonable	[17%; 9%; 10%]
High	[12%; 3%; 3%]

PHC: primary health care; BHU: basic health units; SD: standard deviation;

CRS-SMS / SP: Regional Health Coordination-São Paulo Municipal Health Deparment; FVG: fruit and vegetables and greens; FGBP: *Guia Alimentar para a População Brasileira* (Food Guidelines for the Brazilian Population); BRF: Brazilian regional foods; CFLV: in the kitchen with fruits, vegetables and greens.

^a^ Reference 2020: R$1,045.00.

^b^ Social worker, occupational therapist, physiotherapist, speech therapist, psychologist, physical educator, Environmental Protection Officer.

^c^ Participants could choose more than one answer.

^d^ Friends, recipe exchange groups, gym.

The EFA was initially performed with a version of the instrument validated by experts, with 43 items. Bartlett’s (5.248; gl = 903; p < 0.001) and KMO (0.91: very good) test of sphericity suggested interpretability of the correlation matrix. The parallel analysis suggested four representative factors for the data, with an explained variance of 54.6%. Some items had insignificant factorial loads and commonalities^25^. After these items were excluded, the instrument was analyzed again. Subsequently, items with cross-factorial loads in the interpretation of factors were removed and the instrument underwent a new analysis. The reduced model of the instrument retained 29 of the 43 items. Bartlett’s (5.301,7; gl = 406; p < 0.001) and KMO (0.91: very good) test of sphericity suggested interpretability of the correlation matrix, with four factors identified in the parallel analysis and explained variance of 64.1%.

The final EHAPS model resulted in a scale of the type Likert, with response options on the frequency of actions centered on HCD attributes, with 29 items^b^. The scale score is determined by the sum of the scores corresponding to the options indicated in each item (“never” = 0, “almost never” = 1, “sometimes” = 2, “almost always” = 3 and “always” = 4). Based on the sum of points of the items, four score ranges were proposed with the following statuses: low HCS (0 to 29 points, equivalent to ≤ 25% of the maximum score); moderately low HCS (30 to 58 points, equivalent to > 25% and ≤ 50% of the maximum score); moderately high HCS (59 to 87 points, corresponding to > 50% and ≤ 75% of the maximum score) and high HCS (88 to 116 points, or > 75% of the maximum score). The interpretation of the final score was graphically presented in a ruler format with color gradation (from intense red, representing low HCS, to intense green, representing high HCS), with instructional messages about the score achieved and encouragement to the development of these skills.

Table 3 shows the sequence of item reduction by EFA. The factorial loads of the retained items, composite reliability indexes and replicability estimates of the factor scores (H-index) are shown in Table 4. Names and descriptions of the construct measured by each factor extracted in the EFA are also reported based on the interpretation of the items retained. These factors were understood as dimensions of home cooking skills assessed by the PHCHCSS.

**Table 3:** Sequence of item reduction by exploratory factor analysis of the Primary Health Care Home Cooking Skills Scale.

Variables/labels	Analysis 1 (43 items)^a^					Analysis 2 (32 items)^b^					Analysis 3 (29 items)				
	Bartlett = 5,248; gl = 903 (p < 0.001)					Bartlett = 5,290. 2; gl = 496 (p < 0.001)					Bartlett = 5,301. 7; gl = 406 (p < 0.001)				
	KMO = 0.91443 (very good)					KMO = 0.91791 (very good)					KMO = 0.91762 (very good)				
	Bootstrap 95%CI KMO = (0.883; 0.885)					Bootstrap 95%CI KMO = (0.899; 0.902)					Bootstrap 95%CI KMO = (0.903; 0.904)				
	Dimensions (factors): 4					Dimensions (factors): 4					Dimensions (factors): 4				
	F1	F2	F3	F4	h^2^	F1	F2	F3	F4	h^2^	F1	F2	F3	F4	h^2^
1. Consider the harvest year of food when buying fruits, vegetables and legumes	0.296	-0.246	0.062	0.356	0.334	-	-	-	-	-	-	-	-	-	-
2. Make a list before I go shopping	0.247	-0.266	-0.028	0.339	0.230	-	-	-	-	-	-	-	-	-	-
3. Consider food prices before buying it	0.091	-0.276	0.050	0.462	0.260	-	-	-	-	-	-	-	-	-	-
4. Organize my time to produce the meals I will consume throughout the week	0.546	-0.152	-0.214	0.301	0.397	-	-	-	-	-	-	-	-	-	-
5. Freeze prepared meals in batches to consume on other days	0.539	0.051	-0.291	0.130	0.273	-	-	-	-	-	-	-	-	-	-
6. Plan the menu considering the use of unconventional parts of food (e.g., skins, stalks, seeds)	**0.736**	-0.144	-0.034	0.024	**0.502**	**0.673**	-0.115	-0.072	0.094	**0.443**	**0.682**	-0.081	-0.035	0.001	**0.418**
7. Determine the amount of food to be purchased based on the number of people eating at home	0.212	-0.351	-0.133	**0.719**	**0.537**	0.172	-0.298	-0.179	**0.785**	**0.528**	0.177	-0.222	-0.135	**0.654**	**0.409**
8. Plan the meals I will consume taking into account the variety of foods (e.g., vegetables, legumes, meats, grains)	**0.477**	-0.217	-0.170	**0.560**	**0.615**	**0.403**	-0.177	-0.194	**0.624**	**0.562**	-	-	-	-	-
9. Check the items I have at home before buying food	0.263	-0.094	-0.093	**0.618**	**0.516**	0.238	-0.012	-0.107	**0.606**	**0.484**	0.248	0.024	-0.069	**0.503**	**0.412**
10. Prepare different recipes from the same ingredients	**0.764**	0.070	0.092	-0.032	**0.672**	**0.765**	0.057	0.049	0.010	**0.670**	**0.773**	0.045	0.029	0.025	**0.671**
11. Create different sauces to vary meals	**0.743**	0.002	0.221	-0.217	**0.567**	**0.757**	-0.042	0.152	-0.127	**0.590**	**0.756**	-0.052	0.137	-0.116	**0.581**
12. Use meal leftovers to prepare a new recipe	**0.835**	0.027	0.002	-0.082	**0.633**	**0.852**	0.021	-0.069	-0.023	**0.654**	**0.861**	0.001	-0.090	-0.004	**0.658**
13. Adapt recipes with the ingredients I have at home	**0.837**	0.118	0.006	-0.059	**0.711**	**0.858**	0.110	-0.045	-0.018	**0.739**	**0.862**	0.093	-0.076	0.017	**0.739**
14. Handle unexpected situations when I am cooking (e.g., preparing a soup with overcooked beans)	**0.772**	0.089	-0.001	0.082	**0.729**	**0.802**	0.065	-0.051	0.132	**0.768**	**0.807**	0.052	-0.080	0.163	**0.776**
15. Preparing stocks from *in natura* ingredients (e.g., fresh vegetables, meat trimmings) to impart flavor to culinary preparations	**0.526**	0.003	0.054	0.143	**0.433**	**0.540**	-0.005	0.006	0.185	**0.442**	**0.558**	0.000	-0.000	0.159	**0.440**
16. Identify when a cake is fully baked	-0.066	0.016	**0.409**	0.491	**0.609**	-0.025	0.027	**0.388**	**0.466**	**0.588**	-	-	-	-	-
17. Thicken culinary preparations with cornstarch without forming lumps	0.093	0.031	**0.352**	0.239	0.375	-	-	-	-	-	-	-	-	-	-
18. Use the correct amount of water to prepare fluffy rice	0.050	-0.004	**0.352**	**0.377**	**0.476**	0.053	-0.031	**0.358**	**0.374**	**0.463**	-	-	-	-	-
19. Cut a chicken into pieces	-0.022	0.018	**0.458**	0.134	0.299	-	-	-	-	-	-	-	-	-	-
20. Correct the acidity of sauces using *in natura* ingredients, like carrots	**0.520**	0.063	0.250	-0.073	**0.439**	**0.534**	-0.003	0.201	-0.017	**0.430**	**0.574**	0.002	0.183	-0.062	**0.432**
21. Prepare homemade tomato sauce	**0.589**	-0.045	0.130	-0.029	**0.405**	**0.585**	-0.107	0.089	0.033	**0.400**	**0.618**	-0.101	0.079	-0.008	**0.407**
22. Cook tough meats, such as beef shank, in liquid to make them softer	0.060	0.042	**0.378**	0.286	**0.435**	0.076	0.047	**0.378**	0.263	**0.424**	0.084	0.054	**0.370**	0.254	**0.418**
23. Fry food without making it greasy	0.018	0.208	0.216	0.177	0.246	-	-	-	-	-	-	-	-	-	-
24. Doing other household chores (e.g., laundry, house cleaning) while cooking	-0.008	**0.802**	-0.060	0.067	**0.647**	-0.110	**0.783**	-0.026	0.142	**0.654**	-0.137	**0.795**	-0.005	0.167	**0.692**
25. Attend to matter on the phone while the pots are on the heat	0.062	**0.858**	-0.148	-0.039	**0.655**	0.030	**0.902**	-0.101	-0.083	**0.720**	0.013	**0.919**	-0.057	-0.096	**0.762**
26. Prepare lunch/dinner from scratch in less than 30 minutes	0.235	**0.582**	0.038	-0.146	**0.401**	0.162	**0.605**	0.054	-0.118	**0.411**	0.193	**0.592**	0.078	-0.158	**0.410**
27. Discard the water in which the beans soaked before cooking it	-0.138	0.146	0.108	**0.443**	0.271	-	-	-	-	-	-	-	-	-	-
28. Replace fresh herbs with dried herbs in cooking preparations by using only my sensory perceptions	**0.403**	0.073	0.025	0.201	0.353	-	-	-	-	-	-	-	-	-	-
29. Adjust the amount of seasonings when tasting food during preparation	-0.005	0.001	-0.019	**0.850**	**0.698**	0.037	0.014	-0.018	**0.826**	**0.709**	0.052	0.008	-0.077	**0.869**	**0.733**
30. Combine foods based on their taste	0.101	-0.012	-0.010	**0.770**	**0.680**	0.138	0.021	-0.040	**0.770**	**0.705**	0.166	0.026	-0.102	**0.802**	**0.731**
31. Differentiate sauces prepared with industrialized vegetable stock from those prepared with natural ingredients by using only the palate	**0.311**	-0.034	0.049	**0.366**	0.394	-	-	-	-	-	-	-	-	-	-
32. Identify how cooked food is according to its consistency (e.g., hard, soft, mushy)	-0.092	0.102	0.079	**0.808**	**0.728**	-0.068	0.100	0.130	**0.753**	**0.720**	-0.062	0.103	0.077	**0.812**	**0.763**
33. Recognize that a white sauce is ready according to its thick texture	0.030	0.058	0.269	**0.424**	**0.457**	0.044	0.047	0.283	**0.412**	**0.460**	0.055	0.049	0.247	**0.442**	**0.471**
34. Identify whether foods are suitable for consumption based on their sensory characteristics (e.g., red color of strawberries, soft texture of avocados, sour smell of spoiled food)	-0.227	-0.062	0.224	**0.818**	**0.680**	-0.207	-0.052	0.231	**0.794**	**0.680**	-0.187	-0.060	0.178	**0.831**	**0.691**
35. Use the pressure cooker alone	-0.216	0.117	**0.557**	0.269	**0.496**	-0.210	0.127	**0.616**	0.194	**0.499**	-0.208	0.110	**0.599**	0.223	**0.490**
36. Prepare a sugar syrup	-0.076	0.008	**0.727**	0.127	**0.602**	-0.055	0.009	**0.729**	0.106	**0.595**	-0.064	0.004	**0.716**	0.137	**0.600**
37. Follow a recipe from start to finish	-0.089	-0.050	**0.811**	0.097	**0.654**	-0.094	-0.042	**0.827**	0.090	**0.672**	-0.103	-0.037	**0.823**	0.100	**0.672**
38. Bake homemade bread	0.141	-0.140	**0.789**	-0.179	**0.523**	0.127	-0.150	**0.760**	-0.129	**0.520**	0.114	-0.145	**0.773**	-0.134	**0.528**
39. Cook food as instructed in a recipe (e.g., firm beans for a salad, medium meats)	0.015	-0.068	**0.902**	-0.012	**0.769**	-0.015	-0.062	**0.906**	0.016	**0.785**	-0.029	-0.049	**0.904**	0.025	**0.788**
40. Roast a whole bird	-0.012	0.060	**0.870**	-0.263	**0.562**	-0.005	0.093	**0.861**	-0.282	**0.563**	-0.026	0.091	**0.879**	-0.264	**0.576**
41. Adjust the amount of ingredients in a recipe for a larger number of people	0.066	0.028	**0.762**	-0.010	**0.646**	0.065	0.032	**0.743**	0.008	**0.639**	0.068	0.036	**0.745**	0.016	**0.657**
42. Convert universal measurements (e.g., gram, kilo, liter) to home measurements (e.g., spoonful, glass, cup)	0.177	-0.058	**0.665**	-0.059	**0.510**	0.169	-0.043	**0.631**	-0.026	**0.500**	0.170	-0.032	**0.646**	-0.050	**0.508**
43. Bake a simple cake without instructions	0.060	-0.019	**0.668**	-0.024	**0.460**	0.095	0.006	**0.615**	-0.014	**0.442**	0.120	0.006	**0.610**	-0.045	**0.434**

KMO: Kaiser Mayer Olkin; CI: confidence interval; F: Factor; h^2^: commonality.

Note: the values in bold are highlighted to easily identify which factor the variable belongs to (factorial loads > 0.3). The values of commonality were also highlighted to identify that the variables meet the recommendations (> 0.4).

^a^ Items with factorial loads > 0.3 (regardless of the observation of double saturation) and commonalities greater than 0.4 were retained. We chose not to exclude items with double factorial loads (crossloading) in this step to ascertain the behavior of the loads of these items after analysis.

^b^ Excluded items with double factorial load or double saturation (crossloading). The ( - ) sign corresponds to the excluded item. Results in *italic* represent double saturation.

**Table 4:** Factorial structure of the Primary Health Care Home Cooking Skills Scale after reduction of items.

(Variable) Label	Creative planning	Multitasking skills	Confidence in cooking skills	Food selection, combination and preparation	Commonality (h^2^)
(V 6) Plan the menu considering the use of unconventional parts of food (e.g., skins, stalks, seeds)	**0.682**	-0.081	-0.035	0.001	**0.418**
(V 10) Prepare different recipes from the same ingredients	**0.773**	0.045	0.029	0.025	**0.671**
(V 11) Create different sauces to vary meals	**0.756**	-0.052	0.137	-0.116	**0.581**
(V 12) Use meal leftovers to prepare a new recipe	**0.861**	0.001	-0.090	-0.004	**0.658**
(V 13) Handling unexpected situations when I am cooking (e.g., preparing a soup with overcooked beans)	**0.862**	0.093	-0.076	0.017	**0.739**
(V 14) Adapt recipes with the ingredients I have at home	**0.807**	0.052	-0.080	0.163	**0.776**
(V 15) Prepare stocks from *in natura* ingredients (e.g., fresh vegetables, meat trimmings) to impart flavor to culinary preparations	**0.558**	0.000	-0.000	0.159	**0.440**
(V 20) Correct the acidity of sauces using *in natura* ingredients, like carrots	**0.574**	0.002	0.183	-0.062	**0.432**
(V 21) Prepare homemade tomato sauce	**0.618**	-0.101	0.079	-0.008	**0.407**
(V 24) Doing other household chores (e.g., laundry, house cleaning) while cooking	-0.137	**0.795**	-0.005	0.167	**0.692**
(V 25) Attend to matter on the phone while the pots are on the heat	0.013	**0.919**	-0.057	-0.096	**0.762**
(V 26) Prepare lunch/dinner from scratch in less than 30 minutes	0.193	**0.592**	0.078	-0.158	**0.410**
(V 22) Cook tough meats, such as beef shank, in liquid to make them softer	0.084	0.054	**0.370**	0.254	**0.418**
(V 35) Use the pressure cooker alone	-0.208	0.110	**0.599**	0.223	**0.490**
(V 36) Prepare a caramel syrup	-0.064	0.004	**0.716**	0.137	**0.600**
(V 37) Follow a recipe from start to finish	-0.103	-0.037	**0.823**	0.100	**0.672**
(V 38) Bake homemade bread	0.114	-0.145	**0.773**	-0.134	**0.528**
(V 39) Cook food as instructed in a recipe (e.g., firm beans for a salad, medium meats)	-0.029	-0.049	**0.904**	0.025	**0.788**
(V 40) Roast a whole bird	-0.026	0.091	**0.879**	-0.264	**0.576**
(V 41) Adjust the amount of ingredients in a recipe for a larger number of people	0.068	0.036	**0.745**	0.016	**0.657**
(V 42) Convert universal measurements (e.g., gram, kilo, liter) into home measurements (e.g., spoonful, glass, cup)	0.170	-0.032	**0.646**	-0.050	**0.508**
(V 43) Bake a simple cake without instructions	0.120	0.006	**0.610**	-0.045	**0.434**
(V 7) Decide the amount of food to be purchased based on the number of people eating at home	0.177	-0.222	-0.135	**0.654**	**0.409**
(V 9) Check the items I have at home before buying food	0.248	0.024	-0.069	**0.503**	**0.412**
(V 29) Adjust the amount of seasonings when tasting food during preparation	0.052	0.008	-0.077	**0.869**	**0.733**
(V 30) Combine foods based on their taste	0.166	0.026	-0.102	**0.802**	**0.731**
(V 32) Identify how cooked food is according to its consistency (e.g., hard, soft, mushy)	-0.062	0.103	0.077	**0.812**	**0.763**
(V 33) Recognize that a white sauce is ready according to its thick texture	0.055	0.049	0.247	**0.442**	**0.471**
(V 34) Identify whether foods are fit for consumption based on their sensory characteristics (e.g., red color of strawberries, soft texture of avocados, sour smell of spoiled food)	-0.187	-0.060	0.178	**0.831**	**0.691**
Composite reliability^a^	**0.909**	**0.819**	**0.913**	**0.877**	-
H-latent	**0.871**	**0.940**	**0.938**	**0.931**	-
H-observed	**0.873**	**0.924**	**0.948**	**0.887**	-

Note: the values in bold are highlighted to easily identify which factor the variable belongs to. The values of commonality were also highlighted to identify that the variables meet the recommendations, as well as the values of composite reliability and H-index.

^a^ Calculation performed on statistical platform: http://www.thestatisticalmind.com/calculators/comprel/composite_reliability.htm

The items retained showed adequate loads in their respective factors. No new patterns of cross loads were found in the reduced model (i.e. items with factorial loads > 0.30 in more than one factor). The composite reliability of the factors was adequate (> 0.70) for all factors. The H-index measure suggested replicable factors in future studies (H > 0.80)^28^.

It should be noted that the factorial structure presented adequate adjustment indexes (χ^2^ = 296, gl = 334,246; p = 0.06; RMSEA = 0.037; IFC = 0.99; TLI = 0.99).

## DISCUSSION

This study reported the development of an instrument to measure the home cooking skills of primary health care professionals in the city of São Paulo. The psychometric methodology proved to be appropriate to analyze the reliability and validity of the PHCHCSS.

Although uncommon in scale development studies, the content validity stage had a lay member in the the expert panel^33^. The inclusion of this member allowed identifying and correcting potential problems in the scale in advance of its application for data collection for exploratory factor analysis^8^. The application of the strict consensus method with two measures (CVRc and k) to quantify the degree of agreement among experts resulted in items with strong content validity. The opinion of experts was considered in other studies that reported instruments for measuring cooking skills^12,34^. However, these studies did not present empirical methods derived from the judgment of experts as evidence of the content validity. The fact that experts give opinions on construct items does not in itself provide relevant information for the validation process^13,28^. Thus, this study stands out regarding the methodological rigor employed for content validity of the PHCHCSS.

The pre-test participants reported adequate usability of the instrument. Five health professionals participated in this stage. Rattray et al.^35^ assert that pilot studies can be conducted with small samples as long as the performance of the analyses is not compromised in any way. Considering that the sample was used to qualitatively evaluate the understanding and deployment of the instrument, the number of pre-test professionals did not create limitations to the study.

Regarding the stage of construct validity and reliability of the PHCHCSS, the parallel analysis suggested a multidimensional instrument with four factors. The multidimensionality of the scale is aligned with the complex nature of the acts of eating and cooking, recognized by the *Guia Alimentar para a População Brasileira*^3^.

The creative planning dimension considers creativity when planning and preparing home-cooked meals *in natura*, minimally processed foods and procedures done in advance to facilitate the act of cooking. A similar finding was observed in the study by Jomori^11^, which considers the creative ability to plan menus and organize meal preparation as skills for individual-centered culinary practice. This dimension is related to the main recommendation of the *Guia Alimentar para a População Brasileira*^3^: “You should always prefer *in natura* or minimally processed foods and culinary preparations to ultra-processed foods”. It is also related to the chapter on understanding and overcoming obstacles to putting this and other recommendations into practice. Cooking procedures done in advance shorten the time spent preparing meals. Given the pace of modern life, this obstacle is more easily overcome when multitasking skills are also put into practice.

The dimension of multitasking skills comprises the ability to perform household tasks simultaneously to culinary practices. If an individual is unable to cook while doing laundry and taking care of children, they may be less likely to prepare a home-cooked meal^36^. Gabe^37^ discusses the influence of the home environment on the quality of the meals consumed, highlighting that there is a gender discrepancy regarding responsibility for household chores, which is reinforced by Mills et al.^38^ These findings provide an opportunity to use the PHCHCSS in studies aimed at analyzing differences in multitasking skills between genders, in order to encourage the fair sharing of responsibilities in the home, which includes preparing meals.

The dimension of confidence regarding cooking skills corresponds to self-sufficiency to employ cooking techniques and utensils. According to Martins^12^, the confidence judgment considers individual performance, which depends on practice and task performed, considered an excellent predictor of behavior to determine how individuals employ their skills. The PHCHCSS reduces misinterpretations about HCS by disregarding questions about confidence to prepare meals based on ready-made and convenience products, which could overestimate the individual’s skills, a recurring problem in international instruments^1^. The cooking confidence scale by Lavelle et al.^34^, for example, includes questions about confidence to “prepare food in a microwave oven, including heating ready-made dishes”.

Finally, the dimension of food selection, combination and preparation refers to the sensory and quantification aspects of food aiming at the adequacy of purchasing and cooking procedures. Similar components, which concern the ability to shop for food, use it in preparations and judge it for quality, are found in the study about food literacy by Vidgen and Gallegos^39^. According to the authors, low food literacy is associated with increased diet-related chronic diseases.

The results of the exploratory factor analysis showed adequate factorial loads and commonalities in all items retained in the instrument^27,28^and they suggest a well-defined latent variable, with dimensions that are likely to be stable in future studies^31^. The adjustment indexes presented validated the model extracted from the analysis and confirm the measured theory, showing a well-defined construct^30^. The reliability of the instrument was also adequate, with satisfactory results of composite reliability. This measure represents a good indicator to evaluate the quality of the structural model of the instrument and is presented as a more robust precision indicator, compared to the alpha coefficient^32^.

Developing evaluation instruments is a complex task, only recommended in the lack of another instrument suitable to the reality being investigated^40^, which is the case in this study.

As an advantage, the PHCHCSS is short, easy to apply and standardized, allowing its use in comparative studies. This instrument summarizes the home cooking skills according to scoreranges easy to interpret, delimited by traffic light colors, based on a diagram suggested by Gabe^37^ to interpret the score of her dietary quality assessment instrument, adopted by the Ministry of Health. It also offers messages on the status of the individual’s home cooking skills, with instructions for encouraging and appreciating these skills. It should be noted, however, that the score of the scale derives from its raw score. Although commonly found in studies of instrument development, the use of this score assumes a subjective definition of classification cut-off points, conferring the same weight for items with different factorial loads. The item response theory is an analysis proposal to overcome this limitation by considering the characteristics of the questionnaire items regarding the ability to differentiate the variable of interest and location in the respective *continuum* and a probabilistic model to estimate and describe the scores^41^. Thus, the item response theory could be used in future studies aiming to improve the score of this instrument, validated by classical methods.

Automation minimized possible errors by the interviewer. The online application of the instrument proved advantageous due to its low cost and ease of access. However, its application on paper has not been studied to verify the occurrence of similar results, a limitation of this study. The printed version would allow access to health professionals working in places with limited internet access or not included digitally.

Another limitation is that a convergent validity study was not conducted. This kind of validity refers to the associations of the PHCHCSS score with external measures, which could confirm whether the scale measures HCS related to food choices recommended by the *Guia Alimentar para a População Brasileira* and could be performed by comparing the scale score with a 24-hour dietary recall or with the score of a food literacy scale. Conducting this validity study would be opportune in future analyses.

Finally, the sample used for exploratory factor analysis was composed of professionals working in primary health care in the city of São Paulo. Despite being the main destination for regional migration in Brazil^42^, the sample from this city may not represent the cultural diversity of food within the national territory. Thus, a cross-cultural adaptation of the instrument for Brazilian macroregions is recommended.

This study is innovative in the context of the recognition of cooking as an emancipatory practice and health promotion. It is understood mastering home cooking skills allows primary health care professionals to bring their scientific knowledge closer to people’s lives and to social practices and knowledge, thereby strengthening the ability of individuals or communities to identify solutions for their daily lives. This instrument will make it possible to reliably ascertain the need for qualification of the workforce for actions to promote healthy and adequate food based on home cooking skills. It also provides opportunities to identify needs for reviewing pedagogical proposals of health courses, to train professionals to work on food sovereignty and the human right to adequate food at the expense of medicalizing practices and guidelines.

## Footnotes

a Teixeira AR, Slater B, Miguel F, Camacho JSP, Mega H. PHCHCSS: PHC Home Cooking Skills Scale: from skills to orientation: an instrument for measuring home cooking skills in Primary Health Care. São Paulo: Department of Nutrition and Public Health, University of São Paulo; 2020 [cited 2020 Nov 7]. Doctoral research project. Available from: https://sites.google.com/view/projetohcdusp/

b Teixeira AR, Camanho JSP, Miguel F, Mega H, Slater B. PHC Home Cooking Skills Scale São Paulo: Department of Nutrition and Public Health, Universidade de São Paulo; 2020 [cited 2020 Dec 14]. Available from: https://drive.google.com/file/d/1ORuIN_xfRcLPkCzki5UbZYAM96MLs_VD/view?usp=sharing

## References

[1] McGowan L, Caraher M, Raats M, Lavelle F, Hollywood L, McDowell D, et al. Domestic cooking and food skills: a review. Crit Rev Food Sci Nutr. 2017;57(11):2412-31. 10.1080/10408398.2015.1072495 26618407

[2] Castro IRR. Challenges and perspectives for the promotion of adequate and healthy food in Brazil. Cad Saude Publica. 2015;31(1):1-3. 10.1590/0102-311XPE010115 25715287

[3] Ministério da Saúde (BR), Secretaria de Atenção Primária à Saúde, Departamento de Atenção Básica. Guia alimentar para a população brasileira. 2. ed. Brasília, DF; 2014 [cited 2020 Dec 7]. Available from: https://bvsms.saude.gov.br/bvs/publicacoes/guia_alimentar_populacao_brasileira_2ed.pdf

[4] Pagliai G, Dinu M, Madarena MP, Bonaccio M, Iacoviello L, Sofi F. Consumption of ultra-processed foods and health status: a systematic review and meta-analysis. Br J Nutr. 2021;125(3):308-18. https://doi.org10.1017/S0007114520002688 10.1017/S0007114520002688 PMC7844609 32792031

[5] Askari M, Heshmati J, Shahinfar H, Tripathi N, Daneshzad E. Ultra-processed food and the risk of overweight and obesity: a systematic review and meta-analysis of observational studies. Int J Obes (Lond). 2020;44(10):2080-91. https://doi.org10.1038/s41366-020-00650-z 10.1038/s41366-020-00650-z 32796919

[6] França CJ, Carvalho VCHS. Estratégias de educação alimentar e nutricional na Atenção Primária à Saúde: uma revisão de literatura. Saude Debate. 2017;41(114):932-48. 10.1590/0103-1104201711421

[7] Menezes MFG, Maldonado LA. Do nutricionismo à comida: a culinária como estratégia metodológica de educação alimentar e nutricional. Rev HUPE. 2015;14(3):82-90. 10.12957/rhupe.2015.19950

[8] Morgado FFR, Meireles JFF, Neves CM, Amaral ACS, Ferreira MEC. Scale development: ten main limitations and recommendations to improve future research practices. Psicol Reflex Crit. 2017;30:3. 10.1186/s41155-016-0057-1 PMC6966966 32025957

[9] Reichenheim M, Bastos JL. O quê, para quê e como? Desenvolvendo instrumentos de aferição em epidemiologia. Rev Saude Publica. 2021;55:40. 10.11606/s1518-8787.2021055002813

[10] Teixeira AR, Bicalho D, Slater B, Lima TM. Systematic review of instruments for assessing culinary skills in adults: what is the quality of their psychometric properties? PLoS One. 2021;16(8):e0235182. 10.1371/journal.pone.0235182 PMC8351978 34370729

[11] Jomori MM, Vasconcelos FAG, Bernardo GL, Uggioni PL, Proença RPC. The concept of cooking skills: a review with contributions to the scientific debate. Rev Nutr. 2018;31(1):119-35. 10.1590/1678-98652018000100010

[12] Martins CA, Baraldi LG, Scagliusi FB, Villar BS, Monteiro CA. Cooking Skills Index: development and reliability assessment. Rev Nutr. 2019;32:e180124. 10.1590/1678-9865201932e180124

[13] Furr RM, Bacharach VR. Psychometrics: an introduction. 2. ed. London (UK): SAGE Publications; 2014.

[14] Coluci MZO, Alexandre NMC, Milani D. Construção de instrumentos de medida na área da saúde. Cienc Saude Colet. 2015;20(3):925-36. 10.1590/1413-81232015203.04332013 25760132

[15] DeVellis RF. Scale development: theory and applications. 4. ed. Thousand Oaks, Ca: SAGE Publications; 2017. (Applied Social Research Methods Series; nº 26).

[16] Lynn MR. Determination and quantification of content validity. Nurs Res. 1986;35(6):382-5. 3640358

[17] Gilbert GE, Prion S. Making sense of methods and measurement: Lawshe’s Content Validity Index. Clin Simul Nurs. 2016;12(12):530-1. 10.1016/j.ecns.2016.08.002

[18] Boulkedid R, Abdoul H, Loustau M, Sibony O, Alberti C. Using and reporting the Delphi method for selecting healthcare quality indicators: a systematic review. PLoS One. 2011;6(6):e20476. 10.1371/journal.pone.0020476 PMC3111406 21694759

[19] Wilson FR, Pan W, Schumsky DA. Recalculation of the critical values for Lawshe’s Content Validity Ratio. Meas Eval Couns Dev. 2012;45(3):197-210. 10.1177/0748175612440286

[20] Landis JR, Koch GG. The measurement of observer agreement for categorical data. Biometrics. 1977;33(1):159-7 843571

[21] Pedrosa I, Suárez-Álvarez J, García-Cueto E. Content validity evidences: theoretical advances and estimation methods. Acción Psicol. 2014;10(2):3-18. 10.5944/ap.10.2.11820

[22] Davis LL. Instrument review: getting the most from a panel of experts. Appl Nurs Res. 1992;5(4):194-7. 10.1016/S0897-1897(05)80008-4

[23] Secretaria Municipal da Saúde de São Paulo, Coordenadoria de Epidemilogia e Informação. Relação dos Estabelecimentos/Serviços da Secretaria Municipal da Saúde por Região/Zona. São Paulo: CEInfo; 2021 [cited 2020 Dec 7]. Available from: https://www.prefeitura.sp.gov.br/cidade/secretarias/upload/saude/arquivos/organizacao/Unid_Munic_Saude_Zona.pdf

[24] Costello AB, Osborne JW. Best practices in exploratory factor analysis: four recommendations for getting the most from your analysis. Pract Assess Res Eval. 2005;10:7. 10.7275/jyj1-4868

[25] Timmerman ME, Lorenzo-Seva U. Dimensionality assessment of ordered polytomous items with parallel analysis. Psychol Methods. 2011;16(2):209-20. 10.1037/a0023353 21500916

[26] Lorenzo-Seva U, Ferrando PJ. Robust Promin: a method for diagonally weighted factor rotation. Liberabit Rev Peru Psicol. 2019;25(1):99-106. 10.24265/liberabit.2019.v25n1.08

[27] Tabachnick B, Fidell L. Using multivariate statistics: a practical approach to using multivariate analyses. 6. ed. Boston (USA): Pearson Education; 2013.

[28] Hair JF Jr, Black WC, Babin BJ, Anderson RE. Multivariate data analysis. 7. ed. Harlow (UK): Pearson Education; 2014.

[29] Lorenzo-Seva U, Ferrando PJ. FACTOR: a computer program to fit the exploratory factor analysis model. Behav Res Methods. 2006;38:88-91. 10.3758/BF03192753 16817517

[30] Brown TA. Confirmatory factor analysis for applied research. 2. ed. New York: Guilford Press; 2015.

[31] Ferrando PJ, Lorenzo-Seva U. Assessing the quality and appropriateness of factor solutions and factor score estimates in exploratory item factor analysis. Educ Psychol Meas. 2018;78(5):762-80. https://doi.org/10.1177%2F0013164417719308 10.1177/0013164417719308 PMC7328234 32655169

[32] Valentini F, Damásio BF. Variância média extraída e confiabilidade composta: indicadores de precisão. Psicol Teor Pesq. 2016;32(2):1-7. 10.1590/0102-3772e322225

[33] Epstein J, Santo RM, Guillemin F. A review of guidelines for cross-cultural adaptation of questionnaires could not bring out a consensus. J Clin Epidemiol. 2015;68(4):435-41. 10.1016/j.jclinepi.2014.11.021 25698408

[34] Lavelle F, McGowan L, Hollywood L, Surgenor D, McCloat A, Mooney E, et al. The development and validation of measures to assess cooking skills and food skills. Int J Behav Nutr Phys Act. 2017;14:118. 10.1186/s12966-017-0575-y PMC5581465 28865452

[35] Rattray J, Jones MC. Essential elements of questionnaire design and development. J Clin Nurs. 2007;16(2):234-43. 10.1111/j.1365-2702.2006.01573.x 17239058

[36] Ternier S. Understanding and measuring cooking skills and knowledge as factors influencing convenience food purchases and consumption. Surg J. 2010;3(2):69-76. 10.21083/surg.v3i2.1122

[37] Gabe KT, Jaime PC. Development and testing of a scale to evaluate diet according to the recommendations of the Dietary Guidelines for the Brazilian Population. Public Health Nutr. 2019;22(5):785-96. 10.1017/S1368980018004123 PMC10260633 30744711

[38] Mills S, White M, Brown H, Wrieden W, Kwasnicka D, Halligan J, et al. Health and social determinants and outcomes of home cooking: a systematic review of observational studies. Appetite. 2017;111:116-34. 10.1016/j.appet.2016.12.022 28024883

[39] Vidgen HA, Gallegos D. Defining food literacy and its components. Appetite. 2014;76:50-9. 10.1016/j.appet.2014.01.010 24462490

[40] Streiner DL, Norman GR, Cairney J. Health measurement scales: a practical guide to their development and use. Oxford (UK): Oxford University Press; 2015.

[41] Santos TSS, Araújo PHM, Andrade DF, Louzada MLC, Assis MAA, Slater B. Duas evidências de validade da ESQUADA e níveis de qualidade da dieta dos brasileiros. Rev Saude Publica. 2021;55:39. http://www.rsp.fsp.usp.br/wp-content/uploads/articles_xml/1518-8787-rsp-55-39/1518-8787-rsp-55-39-pt.x34413.pdf 10.11606/s1518-8787.2021055002397 PMC8352565 34406276

[42] Instituto de Políticas Públicas em Direitos Humanos do Mercosul, Organização Internacional para as Migrações. Migrantes regionais na cidade de São Paulo: direitos sociais e políticas públicas. Cidade Autônoma de Buenos Aires (CABA); 2016 [cited 2020 Dec 7]. Available from: https://cidadeseducadoras.org.br/wp-content/uploads/2018/04/migrantes_regionais_na_cidade_de_sao_paulo.pdf

