# Supplementary material for: Instrument for measuring home cooking skills in primary health care
Source: Rev Saude Publica. 2022 Aug 16;56:78. doi: 10.11606/s1518-8787.2022056003473 (PMC9388068; doi:10.11606/s1518-8787.2022056003473)
Supplement: Translation

# Translation: Instrumento para mensurar habilidades culinárias domésticas na atenção primária à saúde

**Authors:** Aline Rissatto Teixeira, Júlia Souza Pinto Camanho, Flavia da Silva Miguel, Helena Carvalho Mega, Betzabeth Slater

**Affiliations:** I Brasil Universidade de São Paulo. Faculdade de Saúde Pública. Programa de Pós-Graduação em Nutrição em Saúde Pública. São Paulo, SP, Brasil; II Brasil Universidade de São Paulo. Faculdade de Saúde Pública. Departamento de Nutrição. São Paulo, SP, Brasil

**Keywords:** Culinária, Habilidade, Psicometria, Estudos de Validação, Educação Alimentar e Nutricional, Atenção Primária à Saúde

## Abstract

**OBJETIVO:**

Desenvolver e validar instrumento para mensuração de habilidades culinárias domésticas de profissionais de saúde envolvidos com orientações de promoção da alimentação adequada e saudável da atenção primária à saúde.

**MÉTODOS:**

Estudo metodológico com abordagem psicométrica, realizado no município de São Paulo entre janeiro e novembro de 2020, para desenvolvimento e validação de instrumento on-line autoaplicável. Os dados dos 472 participantes foram apresentados por estatística descritiva. A validação de conteúdo foi realizada por julgamento de especialistas utilizando técnica Delphi de dois *rounds* e estatísticas empíricas para evidência de consenso. Empregou-se análise fatorial exploratória para validação de constructo e análise de confiabilidade, analisados índices de ajuste do modelo e fidedignidade composta.

**RESULTADOS:**

O instrumento apresentou validade de conteúdo satisfatória para índices de CVRc e 𝜅 nos dois *rounds* da técnica Delphi. Após análise fatorial, o modelo final da Escala de Habilidades Culinárias Domésticas da Atenção Primária à Saúde apresentou 29 itens com cargas fatoriais adequadas (> 0,3). Os testes de esfericidade de *Bartlett* e *Kaiser-Meyer-Olkin* (KMO) realizados em análise fatorial exploratória sugeriram interpretabilidade na matriz de correlação, a análise paralela indicou quatro domínios e variância explicada de 64,1%. A fidedignidade composta dos fatores foi adequada (> 0,70) e o índice H sugeriu fatores replicáveis em estudos futuros. Todos os índices de ajustes mostraram-se adequados.

**CONCLUSÕES:**

A Escala de Habilidades Culinárias Domésticas da Atenção Primária à Saúde apresentou evidências de validade e confiabilidade. É curta e de fácil aplicação e possibilitará a averiguação de forma fidedigna da necessidade de qualificação da força de trabalho, favorecendo o planejamento de ações e políticas públicas de promoção da alimentação adequada e saudável na atenção primária à saúde.

## INTRODUÇÃO

As habilidades culinárias domésticas (HCD) compreendem ações de planejamento do cardápio, seleção, combinação, corte e cocção dos alimentos, capacidade de realizar tarefas concomitantes ao ato de cozinhar e confiança para práticas culinárias^1^. Estão relacionadas às implicações ambientais e econômicas^2^ e são valorizadas pelo Guia Alimentar para a População Brasileira^3^ como expressão de aspectos culturais e sociais. O documento reconhece a culinária como prática estratégica para promoção da alimentação adequada e saudável (PAAS), visando reduzir a escolha de alimentos ultraprocessados, cujo consumo se associa ao sobrepeso, obesidade, câncer e outras doenças^4,5^. Logo, a valorização do ato de cozinhar deve ser central nas ações de educação alimentar e nutricional^2^.

No Brasil, as orientações de PAAS ocorrem substancialmente no âmbito da atenção primária à saúde (APS), o primeiro nível de atenção e vínculo dos sujeitos de direito com o Sistema Único de Saúde. Profissionais da APS desempenham relevante papel ao promover ações de educação alimentar e nutricional que envolvem práticas culinárias, como divulgação de receitas, oficinas, visitas guiadas a feiras livres, visitas domiciliares e exploração sensorial de alimentos^6^. Tais ações possibilitam ao profissional de saúde articular conhecimentos técnicos ao cotidiano dos sujeitos, portanto, é importante que esse profissional se aproprie das habilidades culinárias domésticas^7^.

O diagnóstico preciso dessas habilidades é essencial para promover qualificação da força de trabalho e planejar ações e políticas públicas de saúde sobre a temática e depende do emprego de instrumentos válidos e confiáveis, baseados em critérios psicométricos robustos^8,9^. Teixeira et al.^10^ identificaram e analisaram criticamente a qualidade psicométrica de 12 instrumentos brasileiros e internacionais que mensuram habilidades culinárias em adultos, cujos atributos psicométricos foram considerados insuficientes, com resultados insatisfatórios frente aos critérios estatísticos ou inadequações metodológicas. Dois dos estudos eram brasileiros: Jomori^11^ realizou adaptação transcultural de um instrumento baseado no programa *Cooking with a Chef*, da Universidade de Clemson, cujos resultados de confiabilidade de parte da escala foram insatisfatórios. Martins et al.^12^ desenvolveram uma escala de confiança culinária para pais de crianças em idade escolar. Os autores avaliaram consistência interna, estabilidade e validade de conteúdo do instrumento, porém não reportaram índices de concordância entre especialistas e procedimentos para validade de constructo.

Fortaleceu-se, assim, a necessidade de desenvolver um novo instrumento de avaliação das habilidades culinárias domésticas, destinado aos profissionais de saúde brasileiros que atuam pela PAAS no contexto da APS, com base em critérios psicométricos que respeitem rigor metodológico para determinação de sua validade e confiabilidade recomendados em literatura científica.

## MÉTODOS

Estudo metodológico com abordagem psicométrica^13^, realizado entre janeiro e novembro de 2020.

Este estudo foi aprovado pelo comitê de ética em pesquisa da Universidade de São Paulo (CAAE 15194819.8.0000.5421, no. 3.502.315) e pela instituição coparticipante da Secretaria Municipal de Saúde de São Paulo (SMS-SP) (no. 3.585.369). Os participantes foram informados dos objetivos do estudo e sigilo dos dados por meio de termo de consentimento livre e esclarecido.

Na fase prototípica criou-se um grupo de trabalho com nove integrantes de ambos os sexos e de diferentes estados brasileiros (São Paulo, Mato Grosso, Pará e Minas Gerais) com formação em nutrição e gastronomia, acadêmicos da Faculdade de Saúde Pública da Universidade de São Paulo (FSP-USP) envolvidos com disciplinas de abordagem culinária e pesquisadores de doutorado com experiência em elaboração e validação de instrumentos de pesquisa para desenvolver sistematicamente o instrumento.

Para a definição dos domínios teóricos e itens da primeira versão do instrumento proposto neste estudo, considerou-se: (a) vivência profissional e experiência culinária do grupo; (b) exploração de referencial teórico sobre HCD^14^; (c) revisão sistemática para identificar e analisar propriedades psicométricas de instrumentos que avaliaram as habilidades culinárias domésticas de adultos^10^. Os domínios, itens e formatos de resposta dos instrumentos identificados nesta revisão foram discutidos pelo grupo de pesquisa para a construção do protótipo.

A construção do conjunto inicial de itens e formatos de resposta da versão prototípica do instrumento, intitulado Escala de Habilidades Culinárias Domésticas da Atenção Primária à Saúde (EHAPS), seguiu as recomendações de qualidade propostas por DeVellis^15^.

A fase seguinte, a psicométrica, foi constituída por três etapas. A primeira etapa contou com a participação de especialistas de diversos níveis profissionais, entre docentes universitários, pesquisadores e profissionais de nutrição e gastronomia do Brasil^16^. Considerou-se suficiente a quantidade entre três e 10 participantes^17^.

A técnica Delphi de dois *rounds*^18^ foi realizada. Especialistas preencheram questionários on-line, com questões semiestruturadas de caracterização sociodemográfica e de avaliação dos itens e domínios teóricos do instrumento construídos na fase prototípica. Apontaram melhorias, inclusão/exclusão de itens, adequação das opções da escala do instrumento, e responderam a uma escala *Likert* de concordância (1 = discordo totalmente e 4 = concordo totalmente), para avaliação de cada item quanto a:

- Clareza: O item foi redigido de forma que o conceito esteja compreensível e expressa adequadamente o que se espera medir?
- Pertinência: O item reflete os conceitos envolvidos no domínio e é adequado para atingir os objetivos propostos?
- Relevância: O item é importante para a construção dos domínios que são o foco da escala de pesquisa?

O primeiro *round* do painel ocorreu entre 26 de março e 29 de abril de 2020 e contou com oito especialistas. O grupo de pesquisa examinou os comentários fornecidos, excluiu itens irrelevantes e não pertinentes, realizou adequações daqueles considerados não claros e incluiu itens sugeridos para melhor abrangência do fenômeno. O instrumento foi reapresentado aos especialistas para avaliação das reestruturações. O segundo *round*, iniciado em 28 de maio de 2020, durou 30 dias e contou com sete especialistas.

As características dos participantes do estudo foram apresentadas por estatística descritiva. A razão crítica de validade de conteúdo (*Critical Content Validity Ratio* – CVRc) foi utilizada para analisar estatisticamente a validade de cada atributo dos itens e domínios^19^e o coeficiente Kappa (k) foi calculado para avaliar a concordância entre especialistas sobre cada item^20^ dos dois *rounds* do painel. Foram retidos itens com CVRc > 0,05^19^ e k ≥ 0,60^20^. O índice de validade de conteúdo (IVC) também foi utilizado para analisar a validade do instrumento como um todo^21^. Resultado > 0,8 foi considerado aceitável^22^.

A segunda etapa foi a fase de pré-teste, em que profissionais de um centro de saúde do município de São Paulo, com características similares à população de pesquisa do projeto, testaram a usabilidade da EHAPS. Os participantes do pré-teste não integraram a amostra de validade de constructo e análise de confiabilidade do instrumento. Eles comentaram sobre possíveis dificuldades de preenchimento do instrumento, clareza e adequação das questões ao objetivo da pesquisa e registraram tempo de resposta.

Na terceira etapa, a validade de constructo e a confiabilidade da EHAPS foram testadas. A escala foi desenvolvida para profissionais envolvidos com a promoção da alimentação adequada e saudável de unidades básicas de saúde (UBS) da SMS-SP. Há 464 UBS no município de São Paulo^23^.

Integraram a amostra os profissionais que registraram consentimento de sua participação. O recrutamento se deu por contato com coordenadorias regionais de saúde, supervisões técnicas de saúde e gestores de UBS para coleta de e-mails de profissionais de interesse. Também foi desenvolvido um site^a^, divulgado em mídias sociais para apresentar e esclarecer o objetivo da pesquisa e recrutar participantes. O número de integrantes da amostra baseou-se nas recomendações de Costello e Osborne^24^, de 10 sujeitos por item do instrumento.

A coleta de dados teve início em 2 de agosto de 2020, com duração de 30 dias. Ao todo, 472 profissionais preencheram um questionário sociodemográfico e a EHAPS de forma on-line. Suas características foram apresentadas por estatística descritiva.

Empregou-se análise fatorial exploratória (AFE) para avaliar a estrutura fatorial da EHAPS. Utilizou-se matriz policórica e método de extração *Robust Diagonally Weighted Least Squares* (RDWLS). A decisão sobre o número de fatores retidos se deu por análise paralela com permutação aleatória dos dados observados^25^ e a rotação utilizada foi a *Robust Promin*^26^. Valores de 60% da variância total explicada, itens com comunalidade ≥ 0,4 e cargas fatoriais ≥ 0,30 foram considerados satisfatórios. Itens com cargas fatoriais cruzadas foram excluídos^27^. Valores de KMO ≥ 0,70 e valores significativos para o índice *Bartlett* representaram medidas de adequação da amostra^28^.

A adequação do modelo foi avaliada por índices de ajuste *Root Mean Square Error of Aproximation* (RMSEA), *Comparative Fit Index* (CFI) e *Tucker-Lewis Index* (TLI). Valores de RMSEA devem ser < 0,08, e valores de CFI e TLI devem ser > 0,90 ou, preferencialmente, 0,95^29,30^.

A estabilidade dos fatores foi avaliada pelo índice H, que avalia quão bem um conjunto de itens representa um fator. Valores de H > 0,80 sugerem uma variável latente bem definida e provavelmente estável em diferentes estudos^31^.

Para testar a confiabilidade, calculou-se a fidedignidade composta (FC), com valores aceitáveis > 0,70^32^.

Todas as análises estatísticas foram realizadas utilizando o programa estatístico Factor, versão 10.10.03^29^.

## RESULTADOS

O Quadro detalha os domínios teóricos e construção de conjunto de itens da versão prototípica da EHAPS com base nas discussões do grupo de pesquisa, exploração de referencial teórico e revisão sistemática. Foram propostos 44 itens para avaliar as habilidades culinárias domésticas de profissionais de saúde da APS, com opções de resposta estruturadas em escala tipo *Likert* de cinco pontos (0 = discordo totalmente e 5 = concordo totalmente).

**Quadro:** Identificação de domínios teóricos e construção de conjunto de itens da versão prototípica da EHAPS.

Domínios teóricos	Referência	Rótulos/itens	Referência
1. Planejamento de compras e do preparo de refeições	BRASIL^3^ (2014); Lavelle et al.^34^ (2017); Ternier^36^ (2010); Kennedy et al. (2019)^a^	*Ao planejar minhas compras e refeições, desempenho satisfatoriamente as seguintes tarefas:*	
		1. Pesquisar a safra dos alimentos ao comprar frutas, verduras e legumes	Adaptado de: Lavelle et al.^34^ (2017)
		2. Fazer uma lista de compras antes de ir ao supermercado	Adaptado de: Lavelle et al.^34^ (2017); Kennedy et al. (2019)^a^
		3. Pesquisar preços de alimentos antes de comprá-los	Adaptado de: Lavelle et al.^34^ (2017); Kennedy et al. (2019)^a^
		4. Comprar alimentos em feiras livres	Dos autores
		5. Organizar-se para preparar as refeições que consumirá ao longo da semana	Adaptado de: Lavelle et al.^34^ (2017); Kennedy et al. (2019)^a^
		6. Congelar refeições preparadas em porções para reduzir o tempo na cozinha	Adaptado de: Lavelle et al.^34^ (2017)
2. Criatividade culinária	Lavelle et al.^34^ (2017); Mills et al.^38^ (2017); Short (2006)^b^; Michaud (2007)^c^; Jomori et al. (2017)^d^	*Considero que sou suficientemente criativo para:*	
		7. Cozinhar diferentes preparações a partir dos mesmos ingredientes	Dos autores
		8. Criar diferentes molhos para salada	Dos autores
		9. Aproveitar sobras de refeições para cozinhar uma nova preparação	Adaptado de: Lavelle et al.^34^ (2017); Michaud (2007)^c^; Jomori et al. (2017)^d^
		10. Aproveitar partes não convencionais de alimentos (exemplos: folhas, cascas, talos, sementes) para preparar receitas	Dos autores
3. Habilidades de preparo e multitarefas	Martins et al.^12^ (2019); Lavelle et al.^34^ (2017); Ternier^36^ (2010); Mills et al.^38^ (2017); Kennedy et al. (2019)^a^; Short (2006)^b^ Hartmann et al. (2013)^e^; Kowalkowska et al. (2018)^f^; Vhrovnik (2012)^g^	*Considero ter habilidades suficientes para:*	
		11. Branquear floretes de brócolis, aplicando choque térmico pelo tempo adequado	Dos autores
		12. Fazer remolho rápido do feijão em água quente, descartando a água após 1 hora	Dos autores
		13. Cortar satisfatoriamente uma cebola em cubos pequenos	Adaptado de: Kennedy et al. (2019)^a^
		14. Preparar caldo de legumes a partir de ingredientes frescos	Dos autores
		15. Verificar quando um bolo atingiu seu completo cozimento utilizando um palito	Adaptado de Martins et al.^12^ (2019); Hartmann et al. (2013)^e^; Kowalkowska et al. (2018)^f^
		16. Espessar preparações com amido sem formar grumos	Dos autores
		17. Medir a quantidade correta de água para preparar um arroz soltinho	Dos autores
		18. Fazer o dessalgue rápido de carne seca em água fervente	Dos autores
		19. Destrinchar um frango sozinho	Dos autores
		20. Corrigir a acidez de molhos a partir de ingredientes *in natura*, como cenoura	Dos autores
		21. Preparar molho de tomate caseiro	Adaptado de Martins et al.^12^ (2019); Hartmann et al. (2013)^e^; Kowalkowska et al. (2018)^f^
		22. Amaciar carnes rígidas como músculo utilizando a técnica de ensopar	Dos autores
		23. Preparar uma feijoada caseira desde o início	Dos autores
		24. Fritar batatas satisfatoriamente, sem que fiquem oleosas	Adaptado de: Lavelle et al.^34^ (2017); Kennedy et al. (2019)^a^ Vhrovnik (2012)^g^
		25. Cozinhar enquanto faz outras tarefas domésticas (ex.: Lavar roupa, limpar a casa)	Dos autores
		26. Resolver uma pendência por telefone enquanto cozinha macarrão	Dos autores
		27. Preparar uma refeição principal (almoço/jantar) em menos de 30 minutos	Adaptado de: Lavelle et al.^34^ (2017)
4. Percepção sensorial	Lavelle et al.^34^ (2017); Mills et al.^38^ (2017); Short (2006)^b^; Vhrovnok (2012)^g^.	*Considero minhas percepções sensoriais adequadas para:*	
		28. Substituir ervas frescas por ervas secas em preparações culinárias utilizando apenas suas percepções sensoriais	Adaptado de: Lavelle et al.^34^ (2017); Kennedy et al. (2019)^a^
		29. Dosar a quantidade de temperos culinários ao experimentar alimentos durante o preparo	Dos autores
		30. Combinar alimentos com base em experiências culinárias prévias	Dos autores
		31. Julgar que uma refeição a base de macarrão ao sugo, suco de melancia e gelatina de morango oferece inadequado apelo visual	Dos autores
		32. Diferenciar molhos preparados com caldo de legumes industrializados daqueles preparados com ingredientes naturais utilizando apenas o paladar	Dos autores
		33. Identificar o ponto da carne grelhada (malpassado, ao ponto, bem passado) utilizando apenas suas percepções de textura. (ex.: ao pressioná-la com uma espátula)	Adaptado de Vhrovnok (2012)^g^
		34. Reconhecer que a farinha utilizada no preparo de um molho branco está devidamente cozida, utilizando apenas o olfato (identificar aroma amendoado)	Dos autores
5. Confiança	Martins et al.^12^ (2019); Lavelle et al.^34^ (2017); Short (2006)^b^; Michaud (2007)^c^; Jomori et al. (2017)^d^; Hartmann et al. (2013)^e^; Kowalkowska et al. (2018)^f^ Barton et al. (2011)^h^	*Sou suficientemente confiante para:*	
		35. Usar a panela de pressão sozinho	Adaptado de Martins et al.^12^ (2019)
		36. Preparar um caramelo para pudim	Dos autores
		37. Seguir uma receita do começo ao fim	Adaptado de Michaud (2007)^c^; Jomori et al. (2017)^d^; Barton et al. (2011)^h^
		38. Preparar um pão caseiro sozinho	Adaptado de: Hartmann et al. (2013)^e^; Kowalkowska et al (2018)^f^
		39. Atingir o ponto desejado de uma carne enquanto grelha	Dos autores
		40. Assar uma ave inteira	Adaptado de: Lavelle et al.^34^ (2017)
		41. Ajustar a quantidade de ingredientes de uma receita para um número maior de pessoas	Dos autores
		42. Converter medidas universais (grama, quilo, litro) em medidas caseiras (colher, copo, xícara)	Dos autores
		43. Preparar um bolo simples sem receita	Adaptado de Martins et al.^12^ (2019); Hartmann et al. (2013)^e^; Kowalkowska et al. (2018)^f^
		44. Lidar com situações inesperadas quando está cozinhando (ex.: transformar chantilly que passou do ponto em manteiga)	Dos autores

EHAPS: Escala de Habilidades Culinárias Domésticas da Atenção Primária à Saúde.

^a^ Kennedy LG, Kichler EJ, Seabrook JA, Matthews JI, Dworatzek PDN. Validity and Reliability of a Food Skills Questionnaire. J Nutr Educ Behav. 2019;51(7):857-864. DOI: https://doi.org/10.1016/j.jneb.2019.02.003

^b^ Short F. Kitchen Secrets: Berg Publishers; 2006.168 p

^c^ Michaud P. Development and evaluation of instruments to measure the effectiveness of a culinary and Nutrition education program. Thesis. Clemson: Clemson University, SC. 2007

^d^ Jomori, MM; Proença, RPdaC; Echevarria-Guanilo, ME; Bernardo, GL; Uggioni, PL; Fernandes, AC. Construct validity of Brazilian cooking skills and healthy eating questionnaire by the known-groups method. Br Food J. 2017, 119(5)00-00. DOI: http://dx.doi.org/10.1108/BFJ-10-2016-0448

^e^ Hartmann C, Dohle S, Siegrist M.Importance of cooking skills for balanced food choices. Appetite. 2013; 65, 125-31. DOI: https://doi.org/10.1016/j.appet.2013.01.016

^f^ Kowalkowska J, Poı´nhos R; Rodrigues S. Cooking skills and socio-demographics among Portuguese university students. Br Food J. 2018, 120(3)563–577.DOI: https://doi.org/10.1108/BFJ-06-2017-0345

^g^ Vrhovnik L. A pilot study for the development of a food skills survey tool. Dissertation. Queen’s University. Kingston, Ontario, Canada, 2012. DOI: https://doi.org/10.1007/s10653-011-9439-6

^h^ Barton KL, Wrieden WL, Anderson AS. Validity and reliability of a short questionnaire for assessing the impact of cooking skills interventions. J Hum Nutr Diet. 2011; 24, 588–595. DOI: https://doi.org/10.1111/j.1365-277X.2011.01180.x

Os instrumentos identificados em revisão sistemática apresentavam dimensões de planejamento, seleção e compra de alimentos e confiança no preparo de alimentos, podendo ou não incluir produtos pré-preparados e de conveniência.

Para a EHAPS, considerou-se como dimensões teóricas de HCD para a construção dos itens iniciais o planejamento de compras e do preparo de refeições, criatividade culinária, uso de percepção sensorial e confiança no preparo de refeições baseadas em alimentos *in natura*, minimamente processados e ingredientes culinários, como preconiza o Guia Alimentar para a População Brasileira. As habilidades multitarefas também foram identificadas como domínio teórico, apontadas na literatura científica como a capacidade de realizar tarefas simultaneamente no ambiente doméstico, representando uma vantagem para o preparo de refeições.

A versão prototípica do instrumento foi submetida à avaliação de conteúdo por especialistas. Os principais resultados do desenvolvimento e validação da EHAPS são apresentados na Figura.

**Figura:** Fluxo do processo de desenvolvimento dos itens e domínios do instrumento.

EHAPS: Escala de Habilidades Culinárias Domésticas da Atenção Primária à Saúde; APS: atenção primária à saúde.

Na fase psicométrica, a primeira etapa foi a de validação do conteúdo. O estudo apresentou tamanho amostral adequado para essa etapa. A taxa de resposta para o primeiro *round* da técnica Delphi foi de 72,7% (8/11) e 87,5% (7/8) para o segundo *round.* A maioria dos especialistas era do sexo feminino (n = 7; 87,5%), com média de idade de 42,3 anos (DP = 9,0). Do total, 37,5% (n = 3) eram especialistas (*latu sensu*), 12,5% (n = 1) mestres, 25% (n = 2) doutores e 12,5% (n = 1) livre docentes. O painel também contou com um participante laico (n = 1; 12,5%) com aperfeiçoamento em cursos livres de gastronomia e 32 horas semanais dedicadas à culinária. Os especialistas eram docentes em universidades públicas (25%) e privadas (12,5%), pesquisadores (12,5%), nutricionistas registrados em serviços de alimentação (37,5%) e culinaristas (12,5%). O tempo de experiência profissional variou de 10 a 33 anos (média = 17,8 anos; DP = 7,9 anos). O tempo médio dedicado às práticas culinárias entre especialistas foi de 12,2 horas semanais (DP = 9,6 horas semanais).

A avaliação dos especialistas resultou na exclusão de sete itens da versão prototípica do instrumento, dois itens transferidos de domínio, três itens revisados quanto à clareza, seis novos itens propostos e alteração da escala de concordância para uma escala de frequência, totalizando 43 itens válidos quanto ao conteúdo. Uma visão geral da análise de validade de conteúdo do instrumento é demonstrada na Tabela 1.

**Tabela 1:** Análise de validade de conteúdo da Escala de Habilidades Culinárias Domésticas da Atenção Primária à Saúde por especialistas.

Round 1: 8 especialistas								
Abrangência^a^	κ^b^ (%)	Ação	Itens^c^	Clareza^a^	Pertinência^a^	Relevância^a^	κ^b^ (%)	Ação
**Domínio 1. Planejamento de compras e preparo de refeições** Ao planejar minhas compras e refeições, desempenho satisfatoriamente as seguintes tarefas:								
0,5	**0,14 (57,1)**	Adicionar itens	Pesquisar a safra dos alimentos ao comprar frutas, verduras e legumes	0,75	1	1	0,83 (91,7)	Válido
			Fazer uma lista de compras antes de ir ao supermercado	0,75	1	1	0,83 (91,7)	Válido
			Pesquisar preços de alimentos antes de comprá-los	0,75	1	0,75	0,67 (83,3)	Válido
			Comprar alimentos em feiras livres	0,75	0,75	0,75	0,5 (75,0)	Excluído pelo autor^d^
			Organizar-se para preparar as refeições que consumirá ao longo da semana	0,75	1	1	0,83 (91,7)	Válido
			Congelar refeições preparadas em porções para reduzir o tempo na cozinha	**0,5**	1	1	0,71 (85,7)	Revisar clareza
**Domínio 2. Criatividade culinária** Considero que sou suficientemente criativo para:								
0,5	**0,14 (57,1)**	Adicionar itens	Cozinhar diferentes preparações a partir dos mesmos ingredientes	1	1	1	1 (100)	
			Criar diferentes molhos para salada	1	1	0,75	0,83 (91,7)	
			Aproveitar sobras de refeições para cozinhar uma nova preparação	1	1	1	1 (100)	Válido
			Aproveitar partes não convencionais de alimentos (exemplos: folhas, cascas, talos, sementes) para preparar receitas	**0,5**	0,75	0,75	**0,38** (69,5)	Transferir para D1 c/ alteração
**Domínio 3. Habilidades de preparo e multitarefas** Considero ter habilidades suficientes para:								
1	1 (100)	Domínio abrangente	Branquear floretes de brócolis, aplicando choque térmico pelo tempo adequado	**0,25**	0,75	**0,5**	**0,19 (59,5)**	Excluído pelo painel
			Fazer remolho rápido do feijão em água quente, descartando a água após 1 hora	**0,25**	**0,5**	**0,5**	**0,07 (53,6)**	Excluído pelo painel
			Cortar satisfatoriamente uma cebola em cubos pequenos	1	1	**0,25**	0,64 (82,1)	Excluído pelo painel
			Preparar caldo de legumes a partir de ingredientes frescos	1	1	1	1 (100)	Válido
			Verificar quando um bolo atingiu seu completo cozimento utilizando um palito	1	1	1	1 (100)	Válido
			Espessar preparações com amido sem formar grumos	0,75	1	1	0,83 (91,7)	Válido
			Medir a quantidade correta de água para preparar um arroz soltinho	1	1	1	1 (100)	Válido
			Fazer o dessalgue rápido de carne seca em água fervente	0,75	0,75	0,75	0,5 (75,0)	Excluído pelo autor^d^
			Destrinchar um frango sozinho	1	1	0,75	0,83 (91,7)	Válido
			Corrigir a acidez de molhos a partir de ingredientes *in natura*, como cenoura	1	1	1	1 (100)	Válido
			Preparar molho de tomate caseiro	1	1	1	1 (100)	Válido
			Amaciar carnes rígidas como músculo utilizando a técnica de ensopar	1	0,75	0,75	0,67 (83,3)	Válido
			Preparar uma feijoada caseira desde o início	1	1	0,75	0,83 (91,7)	Excluído pelo autor^d^
			Fritar batatas satisfatoriamente, sem que fiquem oleosas	1	0,75	1	0,83 (91,7)	Válido
			Cozinhar enquanto faz outras tarefas domésticas (ex.: Lavar roupa, limpar a casa)	1	1	1	1 (100)	Válido
			Resolver uma pendência por telefone enquanto cozinha macarrão	1	0,75	0,75	0,67 (83,3)	Válido
			Preparar uma refeição principal (almoço/jantar) em menos de 30 minutos	0,75	1	1	0,83 (91,7)	Válido
**Domínio 4. Percepção sensorial** Considero minhas percepções sensoriais adequadas para:								
**0,75**	0,5 (75)	Domínio abrangente	Substituir ervas frescas por ervas secas em preparações culinárias utilizando apenas suas percepções sensoriais	0,75	1	1	0,83 (91,7)	Válido
			Dosar a quantidade de temperos culinários ao experimentar alimentos durante o preparo	0,75	1	1	0,83 (91,7)	Válido
			Combinar alimentos com base em experiências culinárias prévias	1	0,75	1	0,83 (91,7)	Válido
			Julgar que uma refeição a base de macarrão ao sugo, suco de melancia e gelatina de morango oferece inadequado apelo visual	**0,25**	**0,5**	**0,5**	**0,07 (53,6)**	Excluído pelo painel
			Diferenciar molhos preparados com caldo de legumes industrializados daqueles preparados com ingredientes naturais utilizando apenas o paladar	1	1	1	1 (100)	Válido
			Identificar o ponto da carne grelhada (malpassado, ao ponto, bem passado) utilizando apenas suas percepções de textura (ex.: ao pressioná-la com uma espátula)	0,75	1	1	0,83 (91,7)	Válido
			Reconhecer que a farinha utilizada no preparo de um molho branco está devidamente cozida, utilizando apenas o olfato (identificar aroma amendoado)	**0,5**	0,75	0,75	**0,38 (69,5)**	Revisar clareza
**Domínio 5. Confiança** Sou suficientemente confiante para:								
**1**	1 (100)	Domínio abrangente	Usar a panela de pressão sozinho	1	1	1	1 (100)	Válido
			Preparar um caramelo para pudim	1	1	0,75	0,83 (91,7)	Válido
			Seguir uma receita do começo ao fim	1	1	1	1 (100)	Válido
			Preparar um pão caseiro sozinho	1	1	1	1 (100)	Válido
			Atingir o ponto desejado de uma carne enquanto grelha	**0,5**	1	1	0,71 (85,7)	Revisar clareza
			Assar uma ave inteira	1	1	1	1 (100)	Válido
			Ajustar a quantidade de ingredientes de uma receita para um número maior de pessoas	1	1	1	1 (100)	Válido
			Converter medidas universais (grama, quilo, litro) em medidas caseiras (colher, copo, xícara)	0,75	1	1	0,83 (91,7)	Válido
			Preparar um bolo simples sem receita	1	1	1	1 (100)	Válido
			Lidar com situações inesperadas quando está cozinhando (ex.: transformar chantilly que passou do ponto em manteiga)	1	1	1	1 (100)	Transferir para D2 s/ alteração
IVC instrumento (Lawshe, 1975)			0,83					
**Round 2: 7 especialistas**								
**Abrangência^a^**	**κ^b^ (%)**	**Ação**	**Itens^c^**	**Clareza^a^**	**Pertinência^a^**	**Relevância^a^**	**κ^b^ (%)**	**Ação**
**Domínio 1. Planejamento de compras e preparo de refeições** Realizo as seguintes tarefas:								
**1**	1 (100)	Domínio abrangente	Considerar a safra dos alimentos ao comprar frutas, verduras e legumes	-	-	-	-	Válido no *Round 1*
			Fazer uma lista antes de ir às compras	-	-	-	-	Válido no *Round 1*
			Considerar os preços de alimentos antes de comprá-los	-	-	-	-	Válido no *Round 1*
			Organizar meu tempo para produzir as refeições que consumirei ao longo da semana	-	-	-	-	Válido no *Round 1*
			Congelar refeições preparadas em porções, para consumir em outros dias	1	-	-	1 (100)	Mantido/válido
			Planejar o cardápio considerando o aproveitamento de partes não convencionais de alimentos (ex.: cascas, talos, sementes)	1	1	**0,71**	0,81 (90,5)	Mantido^e^
			Determinar a quantidade de alimentos a serem comprados com base no número de pessoas que comem em casa^f^	1	1	1	0,81 (90,5)	Válido
			Planejar as refeições que consumirei levando em conta a variedade de alimentos (ex.: verduras, legumes, carnes, grãos)^f^	1	1	1	1 (100)	Válido
			Verificar os itens que tenho em casa antes da compra de alimentos^f^	1	1	1	1 (100)	Válido
**Domínio 2. Criatividade culinária.** Utilizo minha criatividade para:								
**1**	1 (100)	Domínio abrangente	Preparar diferentes receitas culinárias a partir dos mesmos ingredientes	-	-	-	-	Válido no *Round 1*
			Criar diferentes molhos para variar refeições	-	-	-	-	Válido no *Round 1*
			Aproveitar sobras de refeições para preparar uma nova receita culinária	-	-	-	-	Válido no *Round 1*
			Adaptar receitas culinárias com os ingredientes que tenho em casa^f^	1	1	1	1 (100)	Válido
			Lidar com situações inesperadas quando estou cozinhando (ex.: preparar uma sopa com feijão que cozinhou demais)^f^	1	1	**0,71**	0,81 (90,5)	Mantido^e^
**Domínio 3. Habilidades de preparo e multitarefas.** Realizo as seguintes ações:								
**1**	1 (100)	Domínio abrangente	Preparar caldos a partir de ingredientes *in natura* (ex.: legumes frescos, aparas de carnes) para conferir sabor a preparações culinárias	-	-	-	-	Válido no *Round 1*
			Reconhecer quando um bolo atingiu seu completo cozimento	-	-	-	-	Válido no *Round 1*
			Espessar preparações culinárias com amido de milho sem formar grumos	-	-	-	-	Válido no *Round 1*
			Usar a quantidade correta de água para preparar um arroz soltinho	-	-	-	-	Válido no *Round 1*
			Separar um frango em pedaços	-	-	-	-	Válido no *Round 1*
			Corrigir a acidez de molhos utilizando ingredientes *in natura*, como cenoura	-	-	-	-	Válido no *Round 1*
			Preparar molho de tomate caseiro	-	-	-	-	Válido no *Round 1*
			Cozinhar carnes rígidas, como músculo, em líquido para torná-las mais macias	-	-	-	-	Válido no *Round 1*
			Fritar alimentos, sem que fiquem oleosos	-	-	-	-	Válido no *Round 1*
			Fazer outras tarefas domésticas (ex.: lavar roupa, limpar a casa) enquanto cozinho	-	-	-	-	Válido no *Round 1*
			Resolver uma pendência por telefone enquanto as panelas estão no fogo	-	-	-	-	Válido no *Round 1*
			Preparar o almoço/jantar do zero em menos de 30 minutos	-	-	-	-	Válido no *Round 1*
			Descartar a água em que o feijão ficou de molho, antes de cozinhá-lo	1	1	1	1 (100)	Válido
**Domínio 4. Percepção sensorial.** Utilizo minhas percepções sensoriais para:								
		Domínio considerado abrangente no *Round* 1	Substituir ervas frescas por ervas secas em preparações culinárias utilizando apenas minhas percepções sensoriais	-	-	-	-	Válido no *Round 1*
			Ajustar a quantidade de temperos culinários ao experimentar alimentos durante o preparo	-	-	-	-	Válido no *Round 1*
			Combinar alimentos com base em seu sabor	-	-	-	-	Válido no *Round 1*
			Diferenciar molhos preparados com caldo de legumes industrializados daqueles preparados com ingredientes naturais utilizando apenas o paladar	-	-	-	-	Válido no *Round 1*
			Identificar o ponto de cozimento dos alimentos de acordo com a sua consistência (ex.: duro, macio, mole)	-	-	-	-	Válido no *Round 1*
			Reconhecer que um molho branco está pronto de acordo com sua textura espessa	1	-	-	1 (100)	Mantido/válido
			Identificar se os alimentos estão próprios para o consumo a partir de suas características sensoriais (ex.: cor vermelha de morangos, textura macia de abacates, cheiro azedo de comida estragada)	1	1	1	1 (100)	Válido
**Domínio 5. Confiança.** Sinto-me confiante para:								
		Domínio considerado abrangente no *Round* 1	Usar a panela de pressão sozinho	-	-	-	-	Válido no *Round 1*
			Preparar uma calda de açúcar	-	-	-	-	Válido no *Round 1*
			Seguir uma receita culinária do começo ao fim	-	-	-	-	Válido no *Round 1*
			Preparar um pão caseiro	-	-	-	-	Válido no *Round 1*
			Cozinhar os alimentos de acordo com o ponto indicado na receita culinária (ex.: feijões mais firmes para salada, carnes ao ponto)	1			1 (100)	Mantido/válido
			Assar uma ave inteira	-	-	-	-	Válido no *Round 1*
			Ajustar a quantidade de ingredientes de uma receita culinária para um número maior de pessoas	-	-	-	-	Válido no *Round 1*
			Converter medidas universais (ex.: grama, quilo, litro) em medidas caseiras (ex.: colher, copo, xícara)	-	-	-	-	Válido no *Round 1*
			Preparar um bolo simples sem instruções	-	-	-	-	Válido no *Round 1*
IVC instrumento (Lawshe, 1975)			0,97					

IVC: índice de validade de conteúdo.

^a^ *Round 1:* CVRc: 8 especialistas = p = 0,05 = 0,693; *Round 2:* CVRc: 7 especialistas = p = 0,05 = 0,741^19^.

^b^ O coeficiente de *Kappa (*κ*)* para concordância entre especialistas foi realizado com auxílio de calculadora disponível em: http://justusrandolph.net/kappa/.

^c^ Escala de respostas inicial do instrumento correspondia ao tipo *Likert* para concordância, sendo: (1) Discordo fortemente, (2) Discordo, (3) Nem concordo, nem discordo, (4) Concordo, (5) Concordo fortemente. Esta escala de respostas foi alterada para frequência por sugestão de especialistas após *Round 1.* Especialistas relataram haver dificuldade, por parte dos respondentes, no que diz respeito à compreensão de escalas de concordância. Além disso escalas de concordância podem indicar a percepção do indivíduo em relação às suas habilidades, porém não refletem, necessariamente, os comportamentos que possibilitam mensurar, de fato, tais habilidades. Neste caso a adequação para uma escala de frequência mostrou ser mais coerente com o objetivo deste instrumento. A alteração foi aprovada por especialistas participantes no *Round 2*. A nova escala é apresentada a seguir: (1) Nunca, (2) Quase nunca, (3) Às vezes, (4) Quase sempre, (5) Sempre. Autores realizaram as alterações mínimas aos rótulos e questões do instrumento (sem alterar os objetivos dos mesmos) para torná-los coerentes com a nova escala de resposta. Tais alterações foram também aprovadas por especialistas.

^d^ Itens excluídos pelos pesquisadores com anuência do grupo de especialistas mediante consulta em segunda etapa do painel. O item *Comprar alimentos em feiras livres* foi excluído tendo em vista que a aquisição de alimentos pode ser feita em outros locais de compra, não se limitando às feiras livres, e que a temática foi contemplada em outros itens do instrumento; item *Fazer o dessalgue rápido de carne seca em água fervente* excluído tendo em vista que o propósito do instrumento é mensurar as habilidades culinárias para o preparo de refeições do dia a dia ( o consumo de carne seca não é realizado cotidianamente no município de São Paulo) e, considerando o rótulo de frequência, poderia haver viés de interpretação por parte do respondente (o respondente poderia indicar a frequência com que prepara carne seca e não se realiza o dessalgue); item *Preparar uma feijoada caseira desde o início* excluído tendo em vista que o propósito do instrumento é mensurar as habilidades culinárias para o preparo de refeições do dia a dia (a feijoada, por mais que faça parte da culinária típica brasileira, não é preparada cotidianamente no município de São Paulo) e, considerando o rótulo de frequência, poderia ocorrer viés de interpretação por parte do respondente (o respondente poderia indicar a frequência com que prepara feijoada e não se a produz do zero).

^e^ Justificativa não apresentada pelo especialista ou não aceita pelo autor. Apesar de CVRc ligeiramente inferior ao limite crítico de referência para o atributo de relevância^19^, o item foi mantido tendo em vista o valor de concordância entre especialistas quanto ao item e visando assegurar a abrangência do domínio. Optou-se por manter o item e averiguar seu comportamento em análise fatorial.

^f^ Itens novos, submetidos à análise de validade de conteúdo (clareza, pertinência, relevância e concordância entre especialistas) no segundo *Round* do Painel de Especialistas. As temáticas compreendidas nos itens adicionados contemplaram sugestões de especialistas no primeiro *Round,* visando ampliar a abrangência dos domínios.

A segunda etapa, de pré-teste, contou com a participação de cinco profissionais de um centro de saúde do município de São Paulo. A pandemia por covid-19 representou uma dificuldade para recrutamento, diante da demanda intensificada por atendimentos em UBS. Compuseram a amostra mulheres que atuavam como nutricionistas (n = 3; 60%), psicólogas (n = 1; 20%) e enfermeiras (n = 1; 20%). Esta amostra não integrou a análise de validade e confiabilidade do instrumento. Participantes relataram facilidade de acesso ao instrumento pelo computador, compreensão das perguntas e opções de resposta, com sugestão de ampliação de fonte das questões, o que foi adotado pelo grupo de pesquisa. O tempo médio de registro de respostas foi de 15 minutos.

Na terceira etapa realizou-se a validade de constructo e a análise de confiabilidade. O estudo apresentou tamanho amostral adequado para essa etapa. A Tabela 2 mostra as características dos 472 profissionais de saúde das APS participantes.

**Tabela 2:** Características dos profissionais de saúde participantes do estudo de validade de constructo e confiabilidade da Escala de Habilidades Culinárias Domésticas da Atenção Primária à Saúde.

Variável	Profissionais APS
	UBS (n = 472)
	Média (DP) [%]
CRS-SMS/SP	
Norte	[9%]
Sul	[38%]
Sudeste	[11%]
Centro	[3%]
Leste	[33%]
Oeste	[7%]
Idade (anos)	38 (9)
Raça/cor	
Branca	[48%]
Amarela	[3%]
Parda	[33%]
Preta	[15%]
Sexo	
Feminino	[90%]
Masculino	[10%]
Gênero	
Mulher cisgênero	[83%]
Mulher transgênero	[1%]
Homem cisgênero	[9%]
Prefere não informar	[7%]
Estado civil	
Solteiro(a)	[36%]
Casado(a)	[45%]
União estável	[11%]
Divorciado(a)	[7%]
No. Moradores agregados/dependentes	
Mora sozinho	[9%]
Um	[25%]
Dois	[28%]
Três	[23%]
Quatro	[8%]
Cinco	[6%]
Seis ou mais	[1%]
Renda média familiar (salários mínimos)^a^	
Nenhuma	[1%]
< 1	[2%]
1–3	[29%]
3–6	[22%]
6–9	[19%]
9–12	[11%]
12–15	[7%]
> 15	[9%]
Profissão/área de atuação	
Enfermagem	[33%]
Agente comunitário de saúde	[25%]
Medicina	[10%]
Nutricionista	[10%]
Odontologia/saúde bucal	[5%]
Farmácia	[4%]
Outros^b^	[9%]
Escolaridade	
Ensino médio	[21%]
Ensino técnico	[12%]
Bacharelado	[14%]
Pós-graduação (*Lato sensu*)	[48%]
Pós-graduação (*Stricto sensu*)	[5%]
Tempo de consulta (min)	
≤ 15	[35%]
16–30	[42%]
31–45	[16%]
46–60	[5%]
≥ 61	[2%]
Formação em gastronomia/culinária	
Sim	[3%]
Não	[97%]
Experiência profissional prévia com culinária	
Sim	[15%]
Não	[85%]
Interesse por habilidades culinárias na APS	
Sim	[90%]
Não	[10%]
Orienta habilidades culinárias na APS	
Sempre	[28%]
Às vezes	[60%]
Nunca	[12%]
Fontes de consulta sobre habilidades culinárias^c^	
Nenhum	[2%]
Família	[49%]
Livros	[44%]
Revistas/internet/programas	[77%]
Cursos livres/formação profissional	[15%]
Matriciamento/nutricionista da UBS/ Educação continuada na UBS/ Guia Alimentar	[6%]
Outros^d^	[1%]
Deslocamento percorrido para compra de FLV (km)	
≤ 1	[44%]
1–2	[26%]
2–3	[15%]
3–4	[5%]
4–5	[4%]
> 5	[6%]
Compra alimentos para o lar	
Sim	[86%]
Não	[14%]
Refeições fora do lar (no lar/semana)	
0	[11%]
< 2	[49%]
2–4	[22%]
4–6	[13%]
> 6	[6%]
Tempo dedicado à culinária (horas/semana)	6:25 (7:29)
Grau de conhecimento [GAPB; ARB; CFLV]	
Nenhum	[47%; 70%; 72%]
Baixo	[24%; 18%; 15%]
Razoável	[17%; 9%; 10%]
Elevado	[12%; 3%; 3%]

APS: atenção primária à saúde; UBS: unidade básica de saúde; DP: desvio padrão; CRS-SMS/SP: Coordenadoria Regional de Saúde – Secretaria Municipal de Saúde de São Paulo; FLV: frutas, legumes e verduras; GAPB: guia Alimentar para a População Brasileira; ARB: alimentos regionais brasileiros; CFLV: na cozinha com as frutas, legumes e verduras.

^a^ Referência 2020: R$ 1.045,00.

^b^ Assistente social, terapeuta ocupacional, fisioterapeuta, fonoaudiólogo, psicólogo, educador físico, agente de proteção ambiental.

^c^ Participantes puderam optar por mais de uma resposta.

^d^ Amigos, grupos de troca de receitas, academia.

A AFE foi inicialmente realizada com versão do instrumento validada por especialistas, com 43 itens. Os testes de esfericidade de *Bartlett* (5.248; gl = 903; p < 0,001) e KMO (0,91: muito bom) sugeriram interpretabilidade da matriz de correlação. A análise paralela sugeriu quatro fatores representativos para os dados, com variância explicada de 54,6% e alguns itens apresentaram cargas fatoriais e comunalidades insignificantes^25^. Após exclusão desses itens, o instrumento foi novamente analisado. Na sequência, itens com cargas fatoriais cruzadas na interpretação de fatores foram removidos e o instrumento foi submetido a nova análise. O modelo reduzido do instrumento manteve 29 dos 43 itens. Os resultados dos testes de esfericidade de *Bartlett* (5.301,7; gl = 406; p < 0,001) e KMO (0,91: muito bom) sugeriram interpretabilidade da matriz de correlação, com quatro fatores identificados na análise paralela e variância explicada de 64,1%.

O modelo final da EHAPS resultou em uma escala do tipo *Likert*, com opções de resposta sobre a frequência de ações centradas em atributos de HCD, com 29 itens^b^. O escore da escala é determinado pela soma das pontuações correspondentes às opções assinaladas em cada item (“nunca” = 0, “quase nunca” = 1, “às vezes” = 2, “quase sempre” = 3 e “sempre” = 4). A partir da somatória de pontos dos itens, foram propostas quatro faixas de escore com os seguintes status: HCD baixas (0 a 29 pontos, equivalente à ≤ 25% da pontuação máxima); HCD moderadamente baixas (30 a 58 pontos, equivalente a > 25% e ≤ 50% da pontuação máxima); HCD moderadamente altas (59 a 87 pontos, correspondente a > 50% e ≤ 75% da pontuação máxima) e HCD altas (88 a 116 pontos, ou > 75% da pontuação máxima). A interpretação do escore final foi graficamente apresentada em formato de régua com gradação de cores (do vermelho intenso, representando HCD baixas, ao verde intenso, representando HCD altas), com mensagens instrucionais sobre o escore atingido e estímulo ao desenvolvimento dessas habilidades.

A Tabela 3 apresenta a sequência de redução de itens por meio de AFE. As cargas fatoriais dos itens retidos, índices de fidedignidade composta e estimativas de replicabilidade dos escores fatoriais (H-index) são observados na Tabela 4. Também são reportados nomes e descrições do constructo medido por cada fator extraído na AFE com base na interpretação dos itens retidos. Esses fatores foram entendidos como dimensões de habilidades culinárias domésticas avaliadas pela EHAPS.

**Tabela 3:** Sequência de redução de itens por meio de análise fatorial exploratória da Escala de Habilidades Culinárias Domésticas da Atenção Primária à Saúde.

Variáveis/rótulos	Análise 1 (43 itens)^a^					Análise 2 (32 itens)^b^					Análise 3 (29 itens)				
	Bartlett = 5.248; gl = 903 (p < 0,001)					Bartlett = 5.290,2; gl = 496 (p < 0,001)					Bartlett = 5.301,7; gl = 406 (p < 0,001)				
	KMO = 0,91443 (muito bom)					KMO = 0,91791 (muito bom)					KMO = 0.91762 (muito bom)				
	Bootstrap IC95% KMO = (0,883; 0,885)					Bootstrap IC95% KMO = (0,899; 0,902)					Bootstrap IC95% KMO = (0,903; 0,904)				
	Dimensões (fatores): 4					Dimensões (fatores): 4					Dimensões (fatores): 4				
	F1	F2	F3	F4	h^2^	F1	F2	F3	F4	h^2^	F1	F2	F3	F4	h^2^
1. Considerar a safra dos alimentos ao comprar frutas, verduras e legumes	0,296	-0,246	0,062	0,356	0,334	-	-	-	-	-	-	-	-	-	-
2. Fazer uma lista antes de ir às compras	0,247	-0,266	-0,028	0,339	0,230	-	-	-	-	-	-	-	-	-	-
3. Considerar os preços de alimentos antes de comprá-los	0,091	-0,276	0,050	0,462	0,260	-	-	-	-	-	-	-	-	-	-
4. Organizar meu tempo para produzir as refeições que consumirei ao longo da semana	0,546	-0,152	-0,214	0,301	0,397	-	-	-	-	-	-	-	-	-	-
5. Congelar refeições preparadas em porções, para consumir em outros dias	0,539	0,051	-0,291	0,130	0,273	-	-	-	-	-	-	-	-	-	-
6. Planejar o cardápio considerando o aproveitamento de partes não convencionais de alimentos (ex.: cascas, talos, sementes)	**0,736**	-0,144	-0,034	0,024	**0,502**	**0,673**	-0,115	-0,072	0,094	**0,443**	**0,682**	-0,081	-0,035	0,001	**0,418**
7. Determinar a quantidade de alimentos a serem comprados com base no número de pessoas que comem em casa	0,212	-0,351	-0,133	**0,719**	**0,537**	0,172	-0,298	-0,179	**0,785**	**0,528**	0,177	-0,222	-0,135	**0,654**	**0,409**
8. Planejar as refeições que consumirei levando em conta a variedade de alimentos (ex.: verduras, legumes, carnes, grãos)	**0,477**	-0,217	-0,170	**0,560**	**0,615**	**0,403**	-0,177	-0,194	**0,624**	**0,562**	-	-	-	-	-
9. Verificar os itens que tenho em casa antes da compra de alimentos	0,263	-0,094	-0,093	**0,618**	**0,516**	0,238	-0,012	-0,107	**0,606**	**0,484**	0,248	0,024	-0,069	**0,503**	**0,412**
10. Preparar diferentes receitas culinárias a partir dos mesmos ingredientes	**0,764**	0,070	0,092	-0,032	**0,672**	**0,765**	0,057	0,049	0,010	**0,670**	**0,773**	0,045	0,029	0,025	**0,671**
11. Criar diferentes molhos para variar refeições	**0,743**	0,002	0,221	-0,217	**0,567**	**0,757**	-0,042	0,152	-0,127	**0,590**	**0,756**	-0,052	0,137	-0,116	**0,581**
12. Aproveitar sobras de refeições para preparar uma nova receita culinária	**0,835**	0,027	0,002	-0,082	**0,633**	**0,852**	0,021	-0,069	-0,023	**0,654**	**0,861**	0,001	-0,090	-0,004	**0,658**
13. Adaptar receitas culinárias com os ingredientes que tenho em casa	**0,837**	0,118	0,006	-0,059	**0,711**	**0,858**	0,110	-0,045	-0,018	**0,739**	**0,862**	0,093	-0,076	0,017	**0,739**
14. Lidar com situações inesperadas quando estou cozinhando (ex.: preparar uma sopa com feijão que cozinhou demais)	**0,772**	0,089	-0,001	0,082	**0,729**	**0,802**	0,065	-0,051	0,132	**0,768**	**0,807**	0,052	-0,080	0,163	**0,776**
15. Preparar caldos a partir de ingredientes *in natura* (ex.: legumes frescos, aparas de carnes) para conferir sabor a preparações culinárias	**0,526**	0,003	0,054	0,143	**0,433**	**0,540**	-0,005	0,006	0,185	**0,442**	**0,558**	0,000	-0,000	0,159	**0,440**
16. Reconhecer quando um bolo atingiu seu completo cozimento	-0,066	0,016	**0,409**	0,491	**0,609**	-0,025	0,027	**0,388**	**0,466**	**0,588**	-	-	-	-	-
17. Espessar preparações culinárias com amido de milho sem formar grumos	0,093	0,031	**0,352**	0,239	0,375	-	-	-	-	-	-	-	-	-	-
18. Usar a quantidade correta de água para preparar um arroz soltinho	0,050	-0,004	**0,352**	**0,377**	**0,476**	0,053	-0,031	**0,358**	**0,374**	**0,463**	-	-	-	-	-
19. Separar um frango em pedaços	-0,022	0,018	**0,458**	0,134	0,299	-	-	-	-	-	-	-	-	-	-
20. Corrigir a acidez de molhos utilizando ingredientes *in natura*, como cenoura	**0,520**	0,063	0,250	-0,073	**0,439**	**0,534**	-0,003	0,201	-0,017	**0,430**	**0,574**	0,002	0,183	-0,062	**0,432**
21. Preparar molho de tomate caseiro	**0,589**	-0,045	0,130	-0,029	**0,405**	**0,585**	-0,107	0,089	0,033	**0,400**	**0,618**	-0,101	0,079	-0,008	**0,407**
22. Cozinhar carnes rígidas, como músculo, em líquido para torná-las mais macias	0,060	0,042	**0,378**	0,286	**0,435**	0,076	0,047	**0,378**	0,263	**0,424**	0.084	0,054	**0,370**	0,254	**0,418**
23. Fritar alimentos, sem que fiquem oleosos	0,018	0,208	0,216	0,177	0,246	-	-	-	-	-	-	-	-	-	-
24. Fazer outras tarefas domésticas (ex.: lavar roupa, limpar a casa) enquanto cozinho	-0,008	**0,802**	-0,060	0,067	**0,647**	-0,110	**0,783**	-0,026	0,142	**0,654**	-0,137	**0,795**	-0,005	0,167	**0,692**
25. Resolver uma pendência por telefone enquanto as panelas estão no fogo	0,062	**0,858**	-0,148	-0,039	**0,655**	0,030	**0,902**	-0,101	-0,083	**0,720**	0,013	**0,919**	-0,057	-0,096	**0,762**
26. Preparar o almoço/jantar do zero em menos de 30 minutos	0,235	**0,582**	0,038	-0,146	**0,401**	0,162	**0,605**	0,054	-0,118	**0,411**	0,193	**0,592**	0,078	-0,158	**0,410**
27. Descartar a água em que o feijão ficou de molho, antes de cozinhá-lo	-0,138	0,146	0,108	**0,443**	0,271	-	-	-	-	-	-	-	-	-	-
28. Substituir ervas frescas por ervas secas em preparações culinárias utilizando apenas minhas percepções sensoriais	**0,403**	0,073	0,025	0,201	0,353	-	-	-	-	-	-	-	-	-	-
29. Ajustar a quantidade de temperos culinários ao experimentar alimentos durante o preparo	-0,005	0,001	-0,019	**0,850**	**0,698**	0,037	0,014	-0,018	**0,826**	**0,709**	0,052	0,008	-0,077	**0,869**	**0,733**
30. Combinar alimentos com base em seu sabor	0,101	-0,012	-0,010	**0,770**	**0,680**	0,138	0,021	-0,040	**0,770**	**0,705**	0,166	0,026	-0,102	**0,802**	**0,731**
31. Diferenciar molhos preparados com caldo de legumes industrializados daqueles preparados com ingredientes naturais utilizando apenas o paladar	**0,311**	-0,034	0,049	**0,366**	0,394	-	-	-	-	-	-	-	-	-	-
32. Identificar o ponto de cozimento dos alimentos de acordo com a sua consistência (ex.: duro, macio, mole)	-0,092	0,102	0,079	**0,808**	**0,728**	-0,068	0,100	0,130	**0,753**	**0,720**	-0,062	0,103	0,077	**0,812**	**0,763**
33. Reconhecer que um molho branco está pronto de acordo com sua textura espessa	0,030	0,058	0,269	**0,424**	**0,457**	0,044	0,047	0,283	**0,412**	**0,460**	0,055	0,049	0,247	**0,442**	**0,471**
34. Identificar se os alimentos estão próprios para o consumo a partir de suas características sensoriais (ex.: cor vermelha de morangos, textura macia de abacates, cheiro azedo de comida estragada)	-0,227	-0,062	0,224	**0,818**	**0,680**	-0,207	-0,052	0,231	**0,794**	**0,680**	-0,187	-0,060	0,178	**0,831**	**0,691**
35. Usar a panela de pressão sozinho	-0,216	0,117	**0,557**	0,269	**0,496**	-0,210	0,127	**0,616**	0,194	**0,499**	-0,208	0,110	**0,599**	0,223	**0,490**
36. Preparar uma calda de açúcar	-0,076	0,008	**0,727**	0,127	**0,602**	-0,055	0,009	**0,729**	0,106	**0,595**	-0,064	0,004	**0,716**	0,137	**0,600**
37. Seguir uma receita culinária do começo ao fim	-0,089	-0,050	**0,811**	0,097	**0,654**	-0,094	-0,042	**0,827**	0,090	**0,672**	-0,103	-0,037	**0,823**	0,100	**0,672**
38. Preparar um pão caseiro	0,141	-0,140	**0,789**	-0,179	**0,523**	0,127	-0,150	**0,760**	-0,129	**0,520**	0,114	-0,145	**0,773**	-0,134	**0,528**
39. Cozinhar os alimentos de acordo com o ponto indicado na receita culinária (ex.: feijões mais firmes para salada, carnes ao ponto)	0,015	-0,068	**0,902**	-0,012	**0,769**	-0,015	-0,062	**0,906**	0,016	**0,785**	-0,029	-0,049	**0,904**	0,025	**0,788**
40. Assar uma ave inteira	-0,012	0,060	**0,870**	-0,263	**0,562**	-0,005	0,093	**0,861**	-0,282	**0,563**	-0,026	0,091	**0,879**	-0,264	**0,576**
41. Ajustar a quantidade de ingredientes de uma receita culinária para um número maior de pessoas	0,066	0,028	**0,762**	-0,010	**0,646**	0,065	0,032	**0,743**	0,008	**0,639**	0,068	0,036	**0,745**	0,016	**0,657**
42. Converter medidas universais (ex.: grama, quilo, litro) em medidas caseiras (ex.: colher, copo, xícara)	0,177	-0,058	**0,665**	-0,059	**0,510**	0,169	-0,043	**0,631**	-0,026	**0,500**	0,170	-0,032	**0,646**	-0,050	**0,508**
43. Preparar um bolo simples sem instruções	0,060	-0,019	**0,668**	-0,024	**0,460**	0,095	0,006	**0,615**	-0,014	**0,442**	0,120	0,006	**0,610**	-0,045	**0,434**

KMO: *Kaiser Mayer Olkin*; IC: intervalo de confiança; F: fator; h^2^: comunalidade.

Nota: destaque dos valores em negrito na tabela se dá para que se identifique facilmente a qual fator a variável pertence (carga fatorial > 0,3). Os valores de comunalidade também foram destacados para identificar que as variáveis atendem às recomendações (> 0,4).

^a^ Foram retidos itens com cargas fatoriais > 0,3 (independentemente da observação de dupla saturação) e comunalidades superiores a 0,4. Optou-se por não excluir itens com dupla carga fatorial (*crossloading*) nesta etapa para averiguar o comportamento das cargas desses itens após análise.

^b^ Excluídos itens com dupla carga fatorial ou dupla saturação (*crossloading*). O traço à frente dos valores (-) corresponde ao item excluído. Resultados em *itálico* representam dupla saturação.

**Tabela 4:** Estrutura fatorial da Escala de Habilidades Culinárias Domésticas da Atenção Primária à Saúde, após redução de itens.

(Variável) Rótulo	Planejamento criativo	Habilidades multitarefas	Confiança quanto à capacidade culinária	Seleção, combinação e preparo de alimentos	Comunalidade (h^2^)
(V 6) Planejar o cardápio considerando o aproveitamento de partes não convencionais de alimentos (ex.: cascas, talos, sementes)	**0,682**	-0,081	-0,035	0,001	**0,418**
(V 10) Preparar diferentes receitas culinárias a partir dos mesmos ingredientes	**0,773**	0,045	0,029	0,025	**0,671**
(V 11) Criar diferentes molhos para variar refeições	**0,756**	-0,052	0,137	-0,116	**0,581**
(V 12) Aproveitar sobras de refeições para preparar uma nova receita culinária	**0,861**	0,001	-0,090	-0,004	**0,658**
(V 13) Lidar com situações inesperadas quando estou cozinhando (ex.: preparar uma sopa com feijão que cozinhou demais)	**0,862**	0,093	-0,076	0,017	**0,739**
(V 14) Adaptar receitas culinárias com os ingredientes que tenho em casa.	**0,807**	0,052	-0,080	0,163	**0,776**
(V 15) Preparar caldos a partir de ingredientes *in natura* (ex.: legumes frescos, aparas de carnes) para conferir sabor a preparações culinárias	**0,558**	0,000	-0,000	0,159	**0,440**
(V 20) Corrigir a acidez de molhos utilizando ingredientes *in natura*, como cenoura	**0,574**	0,002	0,183	-0,062	**0,432**
(V 21) Preparar molho de tomate caseiro	**0,618**	-0,101	0,079	-0,008	**0,407**
(V 24) Fazer outras tarefas domésticas (ex.: lavar roupa, limpar a casa) enquanto cozinho	-0,137	**0,795**	-0,005	0,167	**0,692**
(V 25) Resolver uma pendência por telefone enquanto as panelas estão no fogo	0,013	**0,919**	-0,057	-0,096	**0,762**
(V 26) Preparar o almoço/jantar do zero em menos de 30 minutos	0,193	**0,592**	0,078	-0,158	**0,410**
(V 22) Cozinhar carnes rígidas, como músculo, em líquido para torná-las mais macias	0,084	0,054	**0,370**	0,254	**0,418**
(V 35) Usar a panela de pressão sozinho	-0,208	0,110	**0,599**	0,223	**0,490**
(V 36) Preparar uma calda de açúcar	-0,064	0,004	**0,716**	0,137	**0,600**
(V 37) Seguir uma receita culinária do começo ao fim	-0,103	-0,037	**0,823**	0,100	**0,672**
(V 38) Preparar um pão caseiro	0,114	-0,145	**0,773**	-0,134	**0,528**
(V 39) Cozinhar os alimentos de acordo com o ponto indicado na receita culinária (ex.: feijões mais firmes para salada, carnes ao ponto)	-0,029	-0,049	**0,904**	0,025	**0,788**
(V 40) Assar uma ave inteira	-0,026	0,091	**0,879**	-0,264	**0,576**
(V 41) Ajustar a quantidade de ingredientes de uma receita culinária para um número maior de pessoas	0,068	0,036	**0,745**	0,016	**0,657**
(V 42) Converter medidas universais (ex.: grama, quilo, litro) em medidas caseiras (ex.: colher, copo, xícara)	0,170	-0,032	**0,646**	-0,050	**0,508**
(V 43) Preparar um bolo simples sem instruções	0,120	0,006	**0,610**	-0,045	**0,434**
(V 7) Determinar a quantidade de alimentos a serem comprados com base no número de pessoas que comem em casa	0,177	-0,222	-0,135	**0,654**	**0,409**
(V 9) Verificar os itens que tenho em casa antes da compra de alimentos	0,248	0,024	-0,069	**0,503**	**0,412**
(V 29) Ajustar a quantidade de temperos culinários ao experimentar alimentos durante o preparo	0,052	0,008	-0,077	**0,869**	**0,733**
(V 30) Combinar alimentos com base em seu sabor	0,166	0,026	-0,102	**0,802**	**0,731**
(V 32) Identificar o ponto de cozimento dos alimentos de acordo com a sua consistência (ex.: duro, macio, mole)	-0,062	0,103	0,077	**0,812**	**0,763**
(V 33) Reconhecer que um molho branco está pronto de acordo com sua textura espessa	0,055	0,049	0,247	**0,442**	**0,471**
(V 34) Identificar se os alimentos estão próprios para o consumo a partir de suas características sensoriais (ex.: cor vermelha de morangos, textura macia de abacates, cheiro azedo de comida estragada)	-0,187	-0,060	0,178	**0,831**	**0,691**
Fidedignidade composta^a^	**0,909**	**0,819**	**0,913**	**0,877**	-
H-latent	**0,871**	**0,940**	**0,938**	**0,931**	-
H-observed	**0,873**	**0,924**	**0,948**	**0,887**	-

Nota: o destaque dos valores em negrito se dá para que se identifique facilmente a qual fator a variável pertence. Os valores de comunalidade também foram destacados para identificar que a variáveis atendem as recomendações, assim como os valores de fidedignidade composta e índice-H.

^a^ Cálculo realizado em plataforma estatística: http://www.thestatisticalmind.com/calculators/comprel/composite_reliability.htm

Os itens retidos apresentaram cargas adequadas em seus respectivos fatores. Não foram encontrados novos padrões de cargas cruzadas no modelo reduzido (i.e., itens com cargas fatoriais > 0,30 em mais de um fator). A fidedignidade composta dos fatores foi adequada (> 0,70) para todos os fatores. A medida de H-index sugeriu fatores replicáveis em estudos futuros (H > 0,80)^28^.

Cabe destacar que a estrutura fatorial apresentou índices de ajuste adequados (χ^2^ = 296, gl = 334.246; p = 0,06; RMSEA = 0,037; CFI = 0,99; TLI = 0,99).

## DISCUSSÃO

Este estudo reportou o desenvolvimento de um instrumento para mensurar as habilidades culinárias domésticas dos profissionais da atenção primária à saúde do município de São Paulo. A metodologia psicométrica revelou-se apropriada para analisar a confiabilidade e validade da EHAPS.

Apesar de ser uma prática pouco comum em estudos de desenvolvimento de escalas, a etapa de validade de conteúdo contou com um membro laico na composição do painel de especialistas^33^. Essa inclusão permitiu identificar e corrigir potenciais problemas na escala antecipadamente à sua aplicação para coleta de dados para análise fatorial exploratória^8^. A aplicação do método de consenso rigoroso com duas medidas (CVR e k) para quantificar o grau de concordância entre especialistas resultou em itens com alta validade de conteúdo. A opinião de especialistas foi considerada em outros estudos que reportaram instrumentos para medir habilidades culinárias^12,34^. No entanto, esses estudos não apresentaram métodos empíricos derivados do julgamento de especialistas como evidência da validade do conteúdo. O fato de especialistas opinarem sobre itens do constructo não fornece por si só informações relevantes para o processo de validação^13,28^. Assim, este estudo se destaca quanto ao rigor metodológico empregado para validade de conteúdo da EHAPS.

Os participantes do pré-teste apontaram adequada usabilidade do instrumento. Essa etapa contou com a participação de cinco profissionais de saúde. Rattray et al.^35^ afirmam que estudos-piloto podem ser realizados usando pequenas amostras, desde que não haja comprometimentos ao desempenho das análises. Considerando que a amostra foi empregada para avaliar qualitativamente a compreensão e operacionalização do instrumento, o número de profissionais do pré-teste não gerou limitações ao estudo.

Em relação à etapa de avaliação do constructo e confiabilidade da EHAPS, a análise paralela sugeriu um instrumento multidimensional com quatro fatores. A multidimensionalidade da escala está alinhada com a natureza complexa dos atos de comer e cozinhar, reconhecidos pelo Guia Alimentar para a População Brasileira^3^.

A dimensão de planejamento criativo considera a criatividade ao planejar e preparar refeições caseiras baseadas em alimentos *in natura* e minimamente processados e antecipação de procedimentos que facilitem o ato de cozinhar. Achado similar foi observado no estudo de Jomori^11^, que considera a capacidade criativa de planejar menus e organizar a preparação de refeições como habilidades para a prática culinária centradas no indivíduo. Essa dimensão está relacionada à principal recomendação do Guia Alimentar para a População Brasileira^3^: “Prefira sempre alimentos *in natura* ou minimamente processados e preparações culinárias a alimentos ultraprocessados”, e ao capítulo sobre a compreensão e superação de obstáculos para colocar essa e outras recomendações em prática. Antecipar procedimentos culinários possibilita abreviar o tempo de preparo de refeições, diante da vida moderna, obstáculo superado com mais facilidade quando habilidades multitarefas também são colocadas em prática.

A dimensão de habilidades multitarefas compreende a capacidade de realizar tarefas domésticas simultâneas às práticas culinárias. Se o indivíduo for incapaz de cozinhar enquanto lava roupa e cuida dos filhos, poderá ser menos propenso a preparar uma refeição caseira^36^. Gabe^37^ discute a influência do ambiente doméstico sobre qualidade das refeições consumidas, destacando discrepância de responsabilidades quanto às tarefas domésticas relacionadas ao gênero, que é reforçada por Mills et al.^38^ Esses achados oportunizam o emprego da EHAPS em estudos que visem analisar diferenças de habilidades multitarefas entre gêneros, de modo a estimular o justo compartilhamento de responsabilidades no lar, o que inclui o preparo de refeições.

A dimensão de confiança quanto à capacidade culinária corresponde à autossuficiência para empregar técnicas e utensílios culinários. Segundo Martins^12^, o julgamento de confiança considera o desempenho individual, que depende da prática e tarefa executada, considerado um excelente preditor de comportamento para determinar como indivíduos empregam suas habilidades. A EHAPS reduz interpretações errôneas sobre as HCD ao desconsiderar questões sobre confiança para preparar refeições à base de produtos pré-prontos e de conveniência, que poderiam superestimar habilidades do indivíduo, problema recorrente em instrumentos internacionais^1^. A escala de confiança culinária, de Lavelle et al.^34^, por exemplo, inclui questões sobre confiança para “preparar alimentos em forno micro-ondas, incluindo o aquecimento de pratos prontos”.

Por fim, a dimensão de seleção, combinação e preparo de alimentos refere-se aos aspectos sensoriais e de quantificação de alimentos visando adequação de compras e procedimentos culinários. Componentes similares, que versam sobre a capacidade de comprar alimentos, utilizá-los em preparações e julgá-los quanto à qualidade, são encontrados no estudo de Vidgen e Gallegos^39^ sobre *food literacy* (letramento em alimentos). Segundo os autores, o baixo letramento em alimentos está associado ao aumento de doenças crônicas relacionadas à dieta.

Os resultados da análise fatorial exploratória evidenciaram cargas fatoriais e comunalidades adequadas em todos os itens retidos no instrumento^27,28^e sugerem uma variável latente bem definida, com dimensões com boa probabilidade de serem estáveis em estudos futuros^31^. Os índices de ajuste apresentados validaram o modelo extraído da análise e confirmam a teoria mensurada, evidenciando constructo bem definido^30^. A confiabilidade do instrumento também se mostrou adequada, com resultados satisfatórios de fidedignidade composta. Essa medida representa um bom indicador para avaliar a qualidade do modelo estrutural do instrumento e é apresentada como um indicador de precisão mais robusto, comparado ao coeficiente alpha^32^.

Desenvolver instrumentos de avaliação é uma tarefa complexa, sendo apenas incentivada na ausência de outro instrumento adequado à realidade investigada^40^, situação observada neste estudo.

Como vantagem, a EHAPS é curta, fácil de aplicar e padronizada, possibilitando utilização em estudos comparativos. Este instrumento sintetiza as habilidades culinárias domésticas de acordo com faixas de escores de fácil interpretação, delimitadas por cores de semáforo, fundamentada em diagramação sugerida por Gabe^37^ para interpretação do escore de seu instrumento de avaliação de qualidade da dieta, adotado pelo Ministério da Saúde. Oferece, ainda, mensagens sobre o status das habilidades culinárias domésticas do indivíduo, com instruções para estímulo e valorização dessas habilidades. Ressalta-se, porém, que a pontuação da escala deriva de seu escore bruto. Embora comumente observada em estudos de desenvolvimento de instrumentos, a utilização desse escore assume uma definição subjetiva de pontos de corte de classificação, conferindo o mesmo peso para itens com diferentes cargas fatoriais. A teoria de resposta ao item é uma proposta de análise para superar essa limitação ao considerar as características dos itens do questionário quanto à capacidade de discriminar a variável de interesse e localização no respectivo *continuum* e um modelo probabilístico para calcular e descrever os escores^41^. Assim, a teoria de resposta ao item poderia ser empregada em estudos futuros, visando aprimorar o escore deste instrumento, validado por métodos clássicos.

A informatização minimizou possíveis erros do entrevistador. A aplicação on-line do instrumento mostrou-se vantajosa devido ao baixo custo e facilidade de acesso. No entanto, sua aplicação em papel não foi estudada para averiguar a ocorrência de resultados semelhantes, o que representa uma limitação. A versão impressa viabilizaria acesso aos profissionais de saúde atuantes em locais com internet limitada ou não incluídos digitalmente.

Outra limitação corresponde à não realização de estudo de validade convergente. Esse tipo de validade refere-se às associações do escore da EHAPS com medidas externas, o que poderia confirmar se a escala mede HCD relacionadas a escolhas alimentares recomendadas pelo Guia Alimentar para a População Brasileira e poderia ser performado comparando-se o escore da escala com um recordatório de 24 horas ou com o escore de uma escala de *food literacy*. Sua realização seria oportuna em análises futuras.

Finalmente, a amostra utilizada para análise fatorial exploratória foi composta por profissionais que atuam na atenção primária do município de São Paulo. Apesar de se consolidar como principal destino das migrações regionais no Brasil^42^, a amostra dessa cidade pode não representar a diversidade cultural da alimentação no território nacional. Desse modo, sugere-se realizar adaptação transcultural do instrumento para macrorregiões brasileiras.

Este estudo é inovador no contexto de valorização da culinária como prática emancipatória e de promoção de saúde. Compreende-se que a apropriação de habilidades culinárias domésticas por profissionais da atenção primária permite aproximar seu conhecimento científico da vida das pessoas, dos saberes e práticas sociais, fortalecendo a capacidade dos indivíduos ou comunidades de identificar soluções para seu cotidiano. Este instrumento possibilitará averiguar fidedignamente a necessidade de qualificação da força de trabalho para ações de promoção da alimentação adequada e saudável baseadas em habilidades culinárias domésticas. Oportuniza, ainda, identificar necessidades de revisão de propostas pedagógicas dos cursos de saúde, para formar profissionais habilitados a atuar pela soberania alimentar e o direito humano à alimentação adequada em detrimento de práticas e orientações medicalizadoras.

## Footnotes

a Teixeira AR, Slater B, Miguel F, Camanho JSP, Mega H. EHAPS -Escala de Habilidades Culinárias da APS: das habilidades à orientação: instrumento para mensuração de habilidades culinárias domésticas na Atenção Primária à Saúde. São Paulo: Departamento de Nutrição e Saúde Pública, Universidade de São Paulo; 2020 [citado 07 nov 2020]. Projeto de pesquisa de doutorado. Disponível em: https://sites.google.com/view/projetohcdusp/

b Teixeira AR, Camanho JSP, Miguel F, Mega H, Slater B. Escala de Habilidades Culinárias Domésticas da APS. São Paulo: Departamento de Nutrição e Saúde Pública, Universidade de São Paulo; 2020 [citado 14 dez 2020]. Disponível em: https://drive.google.com/file/d/1ORuIN_xfRcLPkCzki5UbZYAM96MLs_VD/view?usp=sharing
